# Biological evaluation of novel 6,9-disubstituted purine analogues in high-grade serous ovarian cancer cell lines

**DOI:** 10.55730/1300-0152.2788

**Published:** 2025-12-18

**Authors:** Duygu ALTIPARMAK, Deren DEMİREL YAVUZ, Pınar KUL KARADENİZLİ, İrem DURMAZ ŞAHİN, Meral TUNÇBİLEK

**Affiliations:** 1 Department of Pharmaceutical Chemistry, Faculty of Pharmacy, Ankara University, Ankara, Turkiye; 2 Research Center for Translational Medicine (KUTTAM), Koç University, İstanbul, Turkiye; 3 Graduate School of Health Sciences, Koç University, İstanbul, Turkiye; 4 Graduate School of Health Sciences, Ankara University, Ankara, Turkiye; 5 School of Medicine, Koç University, İstanbul, Turkiye

**Keywords:** Cytotoxicity, high-grade serous ovarian cancer, apoptosis, cell-cycle arrest, synthesis, purine analogues

## Abstract

**Background/aim:**

High-grade serous ovarian cancer (HGSOC) remains one of the most aggressive forms of ovarian malignancy and frequently shows resistance to conventional therapies. This study aimed to synthesize a novel series of purine analogues, 6-[(4-substituted benzyl amine)/(4-substituted aniline)]-9-cyclopentyl purines, and evaluate their anticancer efficacy against HGSOC cell lines.

**Materials and methods:**

We assessed the biological effects of the synthesized purine analogues on the OVCAR3, OVSAHO, and KURAMOCHI HGSOC cell lines using the sulforhodamine B assay. To investigate the mechanism of action, we conducted flow cytometry and western blot analyses, focusing on DNA replication and apoptosis.

**Results:**

Among the tested compounds, compound **8** showed significant cytotoxic activity with IC_50_ values in the low micromolar range. Preliminary data from flow cytometry and western blot analyses indicated that compound **8** may inhibit DNA replication and induce apoptosis, as reflected by changes in cell viability and cell-cycle progression.

**Conclusion:**

Compound **8** may disrupt key proliferative mechanisms in cancer cells by interfering with DNA synthesis and activating programmed cell death pathways. These findings suggest that compound **8** is a promising lead candidate for further development in ovarian cancer therapeutics.

## 1. Introduction

Cancer remains a major global health burden, with nearly 20 million new diagnoses and 10 million deaths reported in 2022 (Cao et al., 2024). Among gynecological malignancies, ovarian cancer ranks fifth in cancer-related mortality, largely due to late diagnosis and the aggressive nature of high-grade serous ovarian carcinoma (HGSOC) (Kurman and Shih, 2016; Torre et al., 2018). HGSOC is characterized by extensive genomic instability, universal *TP53* mutations, and frequent defects in DNA repair pathways, all contributing to poor prognosis and high relapse rates following platinum-based chemotherapy.

Standard treatment involves cytoreductive surgery followed by platinum agents; however, most patients ultimately develop chemoresistance (Patch et al., 2015). Although poly(ADP-ribose) polymerase (PARP) inhibitors have improved outcomes in *BRCA*-mutated or homologous recombination-deficient tumors, resistance to targeted therapies also emerges, underscoring the need for novel agents that can interfere with DNA replication and enhance existing treatment regimens.

Purine analogues constitute a well-established class of antimetabolite drugs that mimic endogenous purine bases and disrupt nucleotide metabolism, DNA synthesis, and repair (Parker, 2009; Bardal et al., 2011). Clinically used molecules such as 6-mercaptopurine, thioguanine, fludarabine, and cladribine exert cytotoxicity through incorporation into DNA/RNA or the inhibition of key metabolic enzymes (Gandhi and Plunkett, 2002; Jeha et al., 2004). While extensively applied for hematological malignancies, their potential in solid tumors, including ovarian cancer, remains comparatively understudied. However, preclinical evidence suggests that selected purine derivatives can sensitize ovarian cancer cells to DNA-damaging agents and induce replication stress or double-strand breaks, particularly in mismatch repair-deficient settings (Swann et al., 1996; Zaffaroni et al., 1996).

Structural modification of the purine scaffold, especially at the 6, 8, and 9 positions, has enabled the development of compounds with improved selectivity, reduced toxicity, and potential kinase-inhibitory properties (Veselý et al., 1994; Meijer et al., 1997). Such modifications may enhance their activity in cases of solid tumors with high proliferative rates and compromised DNA repair, such as HGSOC.

In this study, we synthesized a series of 6-[(4-substituted benzyl amine)/(4-substituted aniline)]-9-cyclopentyl purine analogues and evaluated their cytotoxic effects in three HGSOC cell lines including OVCAR-3, OVSAHO, and KURAMOCHI. We further investigated the mechanism of action of the most active compound, compound **8**, focusing on cell-cycle perturbation and apoptosis. Our findings revealed compound **8** to be a promising lead analogue that induces S-phase disruption and apoptotic cell death in HGSOC models.

## 2. Results

### 2.1. Chemistry

**Figure 1:**
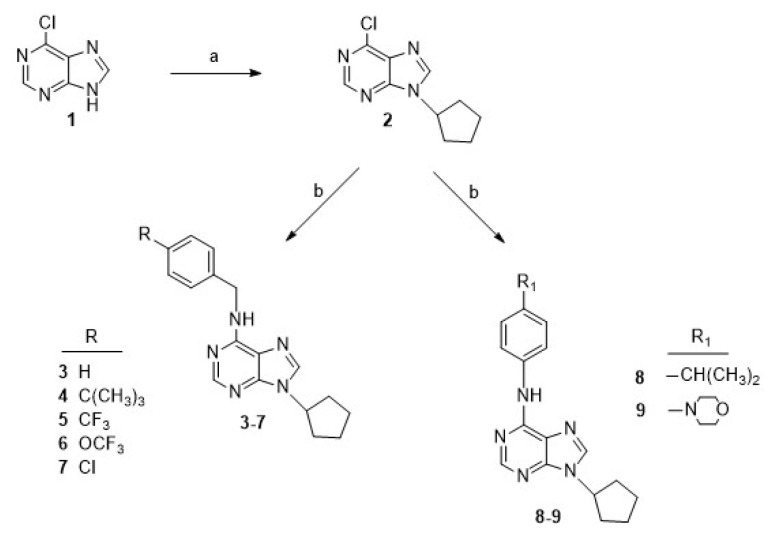
Synthesis of compounds **3**–**9**. Reagents: (a) bromocyclopentane, K_2_CO_3_, DMSO; (b) 4-substituted benzylamines/4-substituted anilines, Et_3_N, EtOH.

We synthesized a series of 6-[(4-substituted benzyl amine)/(4-substituted aniline)]-9-cyclopentyl purine analogues (**3**–**9**) starting from 6-chloropurine (**1**) (Figure 1). First, we alkylated 6-chloropurine (**1**) with cyclopentyl bromide to obtain 6-chloro-9-cyclopentyl-9*H*-purine (**2**) (Tunçbilek et al., 2009). We then carried out a nucleophilic substitution reaction at the 6-position of the purine ring using 4-substituted benzyl amines and 4-substituted anilines in the presence of Et_3_N to produce the final compounds (**3**–**9**). In total, we synthesized seven purine derivatives. We confirmed their purity by thin-layer chromatography and melting point analysis and verified their chemical structures using ^1^H and ^13^C NMR, mass spectrometry, and high-resolution mass spectrometry (HRMS).

### 2.2. In vitro cytotoxicity screening utilizing sulforhodamine B (SRB) assay

**Figure 2:**
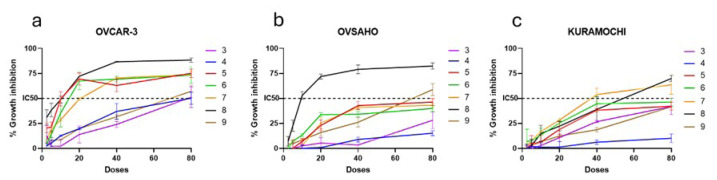
Cytotoxicity evaluation of the synthesized purine analogues. Percent growth inhibition curves were obtained with the NCI SRB assay on HGSOC cell lines OVCAR-3 (**a**), OVSAHO (**b**), and KURAMOCHI (**c**) treated with compounds **3**, **4**, **5**, **6**, **7**, **8**, and **9** for 72 h in increasing concentrations (2.5–80 μM). The experiment was conducted in triplicate (n = 3), and absorbance values were normalized to DMSO negative controls (0.005% v/v for the highest dose and serially diluted respectively) and found to be significant for all (p < 0.001, error bars represent standard deviations).

The cytotoxic activities of seven synthesized purine analogues, labeled as **3**, **4**, **5**, **6**, **7**, **8**, and **9**, were tested for their cytotoxic effects on the OVCAR-3, OVSAHO, and KURAMOCHI HGSOC cell lines utilizing the National Cancer Institute (NCI) SRB assay. Lyophilized chemicals were dissolved in dimethyl sulfoxide (DMSO), obtaining stock concentrations of 20 mM. The NCI SRB assay was performed for doses of 80 μM, 40 μM, 20 μM, 10 μM, 5 μM, and 2.5 μM for 72 h and in triplicate, using a DMSO control for normalization (0.005% v/v for the highest dose and serially diluted respectively). Growth inhibition graphs for each of the three selected cell lines can be seen in Figure 2. Deduction of the IC_50_ values of all compounds for each cell line is summarized in the Table. The results indicated that OVCAR-3 was the most susceptible to cell death caused by the synthesized compounds. Among the seven compounds, **8** had the highest growth inhibition potency with IC_50_ values below 50 μM for all HGSOC cell lines, with the most cytotoxicity observed for the OVCAR-3 cell line. Taking these results into account, OVCAR-3 and compound **8** were chosen to perform further experiments to explore the possible mechanisms of action.

### 2.3. Flow cytometry for cell-cycle analysis with propidium iodide staining

To evaluate the effect of compound **8** on cell-cycle progression, OVCAR-3 cells were treated with 10 μM **8** or an equivalent volume of DMSO (0.001% v/v) for 72 h, followed by staining with propidium iodide (PI). PI intensity reflects cellular DNA content and allows for the discrimination of G0/G1 (2N), S-phase (intermediate DNA content), and G2/M (4N) populations. Cells with fragmented DNA appear as sub-G0/G1 populations.

**Figure 3:**
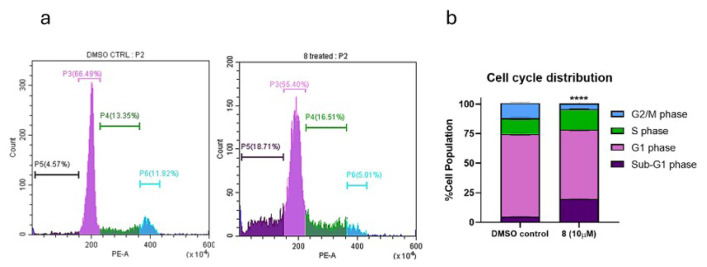
PI staining cell-cycle assay with flow cytometry. Treating HGSOC cell line OVCAR-3 with selected purine analogue **8** (10 μM) had notable effects on the cell cycle compared to the respective DMSO control treatment (0.001%). Histograms (**a**) show the sub-G1 phase in purple, G0/G1 phase in pink, S phase in green, and G2/M phase in blue. Percentages of cell populations are summarized in the stacked-bar graph (**b**), with cell-cycle phases represented with the same colors as in histograms. The four phases show meaningful changes. In the sub-G1 phase, cells increased, indicative of DNA fragmentation, a step of controlled cell death. In the S phase, cells accumulated, suggesting DNA replication stalling or DNA damage accumulation. The G2/M phase showed a decrease in cells, suggesting defects in the transition from the S phase to G2/M. All phase changes were found to be statistically significant (p < 0.001 = ****, error bars represent standard deviations).

Flow cytometry histograms (Figure 3a) and the corresponding quantifications (Figure 3b) demonstrated a significant increase in the sub-G0/G1 fraction following exposure to compound **8**, consistent with apoptotic DNA fragmentation. An accumulation of cells in the S phase was also observed, accompanied by a reduction in the G2/M population. This indicated a disruption in normal cell-cycle progression and suggested the presence of replication stress.

All changes in cell-cycle distribution were statistically significant (n = 3, p < 0.001). As detailed in the next subsection, changes in cyclin E2 levels supported this observed S-phase perturbation.

### 2.4. Western blot results

**Figure 4:**
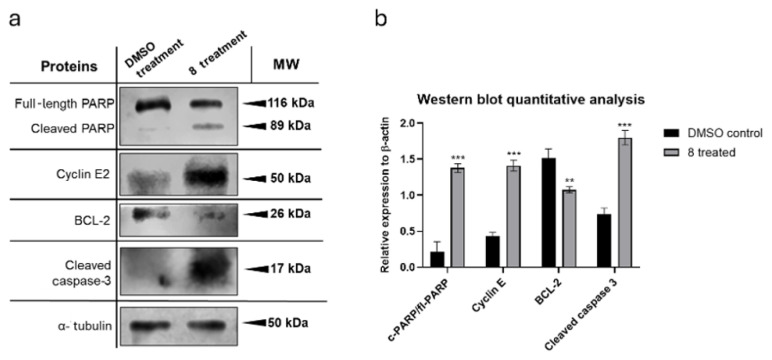
Western blot results. Expressions of proapoptotic proteins cleaved PARP and cleaved caspase-3, antiapoptotic protein BCL-2, and cell-cycle protein cyclin E2 showed significant differences between groups treated with **8** and DMSO control groups (**a**). Changes in the proteins indicated activation of apoptosis and cell-cycle arrest at the S phase or delayed transition from the S phase to G2/M. Quantitative analysis of the blot images is represented as a bar graph (**b**). Statistical significance was observed for all proteins (p < 0.01 = ***, p < 0.05 = **, error bars represent standard deviations).

Western blot analysis revealed increased levels of cleaved PARP in cells treated with compound **8**, indicating activation of the apoptotic machinery. Consistently, cleaved caspase-3 levels were also elevated (Figure 4a). In contrast, the antiapoptotic protein BCL-2 was reduced, supporting the induction of apoptosis by compound **8**.

We additionally examined cyclin E2, a regulator involved in S-phase entry and G2/M transition. Cyclin E2 levels were increased after treatment, consistent with the S-phase accumulation observed in the flow cytometry analysis (Caldon and Musgrove, 2010; Pellarin et al., 2025). These findings align with a model in which compound **8** induces replication stress, preventing efficient progression into mitosis.

Quantification of protein expression (Figure 4b) was performed using open-source ImageJ software. All experiments were conducted using biological replicates and all changes in protein levels were statistically significant (p < 0.05, p < 0.01).

## 3. Discussion

In this study, we synthesized a series of 6,9-disubstituted purine analogues and evaluated their biological activity in HGSOC cell lines. Among the seven synthesized derivatives, compound **8** demonstrated the most potent cytotoxicity, with IC_50_ values below 50 μM across all three models. Notably, its activity surpassed that of carboplatin and olaparib under the same SRB assay conditions, suggesting strong antiproliferative potential in vitro. Although IC_50_ values cannot be directly extrapolated to clinical efficacy, these findings highlight the relevance of compound **8** as a candidate for further preclinical development.

Flow cytometry analysis revealed a significant increase in the sub-G1 population following exposure to compound **8**, indicating apoptotic DNA loss. The concurrent accumulation of cells in the S phase and reduction in the G2/M population suggests perturbation of DNA synthesis and replication stress. This pattern is consistent with the established mechanisms of purine and nucleoside antimetabolites, which disrupt nucleotide metabolism, stall replication forks, and activate cell-death signaling (Gandhi and Plunkett, 2002; Parker, 2009). To avoid overinterpretation, we note that although our data support replication stress, the direct effect on DNA polymerase was not assessed and remains to be explored in future work.

Western blot analysis further confirmed apoptotic induction, as demonstrated by increases in cleaved PARP and cleaved caspase-3, together with downregulation of the antiapoptotic protein BCL-2. PARP cleavage is a hallmark of caspase-3/7 activation during apoptosis (Gobeil et al., 2001). Additionally, the upregulation of cyclin E2, known to accumulate during the S and G2/M phases and associated with replication stress responses, aligns with the cell-cycle arrest observed here (Caldon and Musgrove, 2010; Maya-Mendoza et al., 2018).

Differences in sensitivity to the synthesized compounds among OVCAR-3, OVSAHO, and KURAMOCHI may reflect their distinct genomic backgrounds. KURAMOCHI and OVSAHO are considered to be among the most molecularly faithful HGSOC models, harboring *TP53* mutations and homologous recombination defects similar to those observed in 25% of patient tumors (i.e., HRD-deficient), whereas OVCAR-3 represents a more copy-number-driven, highly proliferative phenotype and is HRD-proficient (Patch et al., 2015; Kurman and Shih, 2016; Torre et al., 2018; Gogola et al., 2019). The heightened sensitivity of OVCAR-3 to compound **8** may therefore reflect increased reliance on nucleotide metabolism or replication machinery. Conversely, the higher IC_50_ observed for KURAMOCHI cells may result from differences in baseline replication stress tolerance or DNA repair capacity. These observations emphasize the importance of cellular context when evaluating purine-based therapeutics.

Preliminary structure–activity relationships in this series provide further insight. Benzyl derivatives containing electron-withdrawing substituents (compounds **5**–**7**) showed moderate activity, whereas the introduction of a more hydrophobic 4-isopropylphenyl group in compound **8** markedly enhanced the potency. In contrast, morpholinophenyl analogue **9** showed weak activity, potentially due to reduced membrane permeability or altered target engagement (Hashmi et al., 2025). Prior studies have shown that modifications at the 6- and 9-positions of purine scaffolds can substantially influence binding affinity, stability, and biological activity (Veselý et al., 1994; Meijer et al., 1997). Our findings align with these reports and suggest that the hydrophobic bulk at the 6-position may enhance functional interactions within the cell (Ragab, 2025).

Collectively, these results demonstrate that compound **8** exerts potent antiproliferative effects in HGSOC through the induction of replication stress, disruption of cell-cycle progression, and activation of apoptotic pathways. These observations are consistent with classical purine antimetabolite behavior (Elion, 1989; Schramm, 2018) and highlight the promise of modified purine scaffolds as potential therapeutic candidates for solid tumors, including ovarian cancer. Future work will focus on identifying the molecular targets of compound **8**, expanding the structure–activity explorations, and assessing efficacy in additional models, including in vivo systems.

## 4. Materials and methods

### 4.1. General

We monitored the progress of the reactions using thin-layer chromatography. To visualize the spots, we used aluminum plates coated with fluorescent indicator F_254_ (Merck, Darmstadt, Germany) and illuminated them with a UV lamp (Camag, Muttenz, Switzerland) at wavelengths of 254 and 366 nm. We recorded the ^1^H and ^13^C NMR spectra using a Varian Mercury-400 FT-NMR device (Varian Inc., Palo Alto, CA, USA) and a Bruker Avance NEO-500 NMR spectrometer (Bruker Corp., Billerica, MA, USA). We used tetramethylsilane (TMS) as the internal standard and DMSO-*d_6_* or CDCl_3_ as solvents. We obtained the mass spectra using the electrospray ionization (ESI+) method on a Waters Micromass ZQ instrument (Waters, Milford, MA, USA). For HRMS, we used methanol as the solvent and collected data with the positive ion ESI+ mode on a Waters LCT Premier XE UPLC/MS TOF system, ensuring that the calculated and observed masses remained within 5 ppm. We measured melting points with a B450 melting point apparatus (BÜCHI Labortechnik AG, Flawil, Switzerland). For column chromatography, we used silica gel 60 with a particle size of 40–63 mm. All chemical reagents used in the synthesis process were purchased from Merck (Darmstadt, Germany), Fluka (Buchs, Switzerland), Sigma-Aldrich (St. Louis, MO, USA), or Acros (Geel, Belgium).

### 4.2. Chemistry

#### 4.2.1. 6-Chloro-9-cyclopentyl-9*H*-purine (2)

A suspension of 6-chloropurine (0.6 g, 3.88 mmol) in DMSO (16 mL) was prepared, and K_2_CO_3_ (0.6 g, 4.34 mmol) was added. The reaction mixture was stirred at 40 °C for 45 min. A solution of cyclopentyl bromide (0.7 mL, 6.53 mmol) in DMF (4 mL) was then added and the mixture was stirred at 40 °C for 48 h. Upon completion, the reaction mixture was treated with water and extracted with ethyl acetate. The combined organic layers were dried over Na_2_SO_4_, filtered, and concentrated under reduced pressure. The crude product was purified by column chromatography using a mixture of CHCl_3_, hexane, and EtOAc (5:1:1) as the eluent to afford compound **2** (0.52 g, 60.5% yield). Mp = 97 °C. ^1^H NMR (DMSO-*d_6_*) δ 1.67–1.72 (m, 2H), 1.84–1.89 (m, 2H), 2.00–2.07 (m, 2H), 2.14–2.20 (m, 2H), 4.94–4.98 (m, 1H), 8.74 (s, 1H), 8.75 (s, 1H). ^13^C NMR (DMSO-*d_6_*) δ 23.56, 31.75, 56.34 (C-cyclopentyl), 131.23, 146.13, 148.96, 151.15, 151.74 (C-purine). MS (ESI+) *m/z*: 223.5 (100%) (M+H), 225.5 (34%) (M+H+2) (Tunçbilek et al., 2009).

#### 4.2.2. General procedure for the synthesis of compounds 3–7

To a suspension of compound **2** (1 equiv.) in absolute ethanol (10 mL), the appropriate 6-substituted benzyl amine derivative (1 equiv.) and triethylamine (3 equiv.) were added. The reaction mixture was refluxed at 80–90 °C for 6–12 h. Upon completion, the mixture was concentrated under reduced pressure and the resulting residue was purified by column chromatography using a mixture of hexane, EtOAc, and isopropanol (20:10:1 or 30:10:1) as the eluent to afford compounds **3**–**7**.

##### 4.2.2.1. *N*-Benzyl-9-cyclopentyl-9H-purin-6-amine (3)

The above procedure was followed with benzylamine to yield **3** (0.11 g, 30% yield). Mp = 107–108 °C. ^1^H NMR (DMSO-*d_6_*) δ 1.62–1.72 (m, 2H), 1.81–1.92 (m, 2H), 1.94–2.03 (m, 2H), 2.08–2.16 (m, 2H), 4.69 (br s, 2H), 4.79–4.87 (m, 1H), 7.17–7.34 (m, 5H), 8.18–8.27 (m, 3H). ^13^C NMR (DMSO-*d_6_*) δ 23.50, 31.87 (C-cyclopentyl), 42.83 (-CH_2_), 55.25 (C-cyclopentyl), 126.50, 127.10, 128.11, 138.02 (C-phenyl), 139.23, 140.20, 151.46, 152.03, 154.32 (C-purine). MS (ESI+) *m/z*: 294.4 (100%) (M+H). HRMS (m/z) [M+H]^+^ Calculated: 294.1719, Found: 294.1711 (Montgomery and Thomas, 1972).

##### 4.2.2.2. *N*-(4-(*tert*-Butyl)benzyl)-9-cyclopentyl-9*H*-purin-6-amine (4)

The above procedure was followed with 4-*tert*-butylbenzylamine to yield **4** (0.19 g, 40% yield). Mp = 183–184 °C. ^1^H NMR (DMSO-*d_6_*) δ 1.66–1.70 (m, 2H), 1.84–1.88 (m, 2H), 1.97–2.02 (m, 2H), 2.11–2.14 (m, 2H), 4.66 (br s, 2H), 4.82–4.85 (m, 1H), 7.25–7.31 (m, 4H), 8.19–8.23 (m, 3H). ^13^C NMR (DMSO-*d_6_*) δ 23.50 (C-cyclopentyl), 31.11, (-CH_3_) 31.88 (C-cyclopentyl), 34.06 (-C), 42.45, 42.83 (-CH_2_), 55.32 (C-cyclopentyl), 119.51, 124.83, 126.91 (C-phenyl), 137.16, 139.17, 148.86, 152.03, 154.34 (C-purine). MS (ESI+) *m/z*: 350.6 (100%) (M+H). HRMS (m/z) [M+H]^+^ Calculated: 350.2345, Found: 350.2344.

##### 4.2.2.3.9-Cyclopentyl-N-(4-(trifluoromethyl)benzyl)-9H-purin-6-amine (5)

The above procedure was followed with 4-(trifluoromethyl)-benzylamine to yield **5** (0.14 g, 29% yield). Mp = 169–171 °C. ^1^H NMR (DMSO-*d_6_*) δ 1.65–1.72 (m, 2H), 1.82–1.89 (m, 2H), 1.94–2.02 (m, 2H), 2.08–2.16 (m, 2H), 4.76–4.86 (m, 3H), 7.53 (d, 2H, *J_o_* = 8.4 Hz), 7.64 (d, 2H, *J_o_* = 7.6 Hz), 8.17 (s, 1H), 8.22 (s, 1H), 8.39 (br s, 1H). ^13^C NMR (DMSO-*d_6_*) δ 23.50, 31.87 (C-cyclopentyl), 42.54 (-CH_2_), 55.38 (C-cyclopentyl), 123.00 C-phenyl), 124.99 (q, -CF_3_), 125.69, 127.39, 127.70 (C-phenyl), 139.45, 145.16, 148.88, 152.01, 154.29 (C-purine). MS (ESI+) *m/z*: 362.5 (100%) (M+H). HRMS (m/z) [M+H]^+^ Calculated: 362.1593, Found: 362.1597.

##### 4.2.2.4. 9-Cyclopentyl-*N*-(4-(trifluoromethoxy)benzyl)-9*H*-purin-6-amine (6)

The above procedure was followed with 4-(trifluoromethoxy)-benzylamine to yield **6** (0.1 g, 21% yield). Mp = 151–153 °C. ^1^H NMR (DMSO-*d_6_*) δ 1.68–1.70 (m, 2H), 1.85–1.91 (m, 2H), 1.96–2.04 (m, 2H), 2.10–2.16 (m, 2H), 4.72 (br s, 2H), 4.81–4.86 (m, 1H), 7.28 (d, 2H, *J_o_* = 8.4 Hz), 7.46 (d, 2H, *J_o_* = 8.8 Hz), 8.20 (s, 1H), 8.23 (s, 1H), 8.36 (br s, 1H). ^13^C NMR (DMSO-*d_6_*) δ 24.01, 32.39 (C-cyclopentyl), 42.80 (-CH_2_), 55.87 (C-cyclopentyl), 119.54, 120.02 (C-phenyl), 121.44 (q, -CF_3_), 129.42, 139.89 (C-phenyl), 140.24, 147.51, 149.40, 152.52, 154.75 (C-purine). MS (ESI+) *m/z*: 378.5 (100%) (M+H). HRMS (m/z) [M+H]^+^ Calculated: 378.1542, Found: 378.1525.

##### 4.2.2.5. *N*-(4-Chlorobenzyl)-9-cyclopentyl-9*H*-purin-6-amine (7)

The above procedure was followed with 4-chlorobenzylamine to yield **7** (0.11 g, 25% yield). Mp = 169–171 °C. ^1^H NMR (DMSO-*d_6_*) δ 1.63–1.70 (m, 2H), 1.80–1.89 (m, 2H), 1.92–2.01 (m, 2H), 2.07–2.15 (m, 2H), 4.65 (br s, 2H), 4.77–4.85 (m, 1H), 7.30–7.35 (m, 4H), 8.17–8.31 (m, 3H). ^13^C NMR (DMSO-*d_6_*) δ 23.50, 31.88 (C-cyclopentyl), 42.17 (-CH_2_), 55.35 (C-cyclopentyl), 128.08, 128.99, 131.06, 139.34 (C-phenyl), 152.01, 154.25 (C-purine). MS (ESI+) *m/z*: 328.4 (100%) (M+H), 330.5 (34%) (M+H+2) (Tunçbilek et al., 2009).

#### 4.2.3. General procedure for the synthesis of compounds 8 and 9

A suspension of compound **2** (1 equiv.) in absolute ethanol (10 mL) was treated with the appropriate 6-substituted aniline (1 equiv.) and triethylamine (3 equiv.). The reaction mixture was refluxed at 80–90 °C for 6–12 h. Upon completion, the mixture was concentrated under reduced pressure, and the resulting residue was purified by column chromatography using a mixture of hexane, EtOAc, and isopropanol (20:10:1 or 30:10:1) as the eluent to afford compounds **8** and **9**.

##### 4.2.3.1. 9-cyclopentyl-*N*-(4-isopropylphenyl)-9*H*-purin-6-amine (8)

The above procedure was followed with 4-isopropylaniline to yield **8** (0.12 g, 27% yield). Mp = 173–175 °C. ^1^H NMR (DMSO-*d_6_*) δ 1.20 (d, 6H), 1.69–1.74 (m, 2H), 1.88–1.92 (m, 2H), 2.01–2.06 (m, 2H), 2.14–2.20 (m, 2H), 2.84–2.87 (m, 1H), 4.88–4.92 (m, 1H), 7.18 (d, 2H, *J_o_* = 8.4 Hz), 7.80–7.83 (m, 2H), 8.35 (s, 1H), 8.36 (s, 1H), 9.73 (s, 1H). ^13^C NMR (DMSO-*d_6_*) δ 23.58 (C-cyclopentyl), 23.99 (-CH_3_), 31.91 (C-cyclopentyl), 32.87 (-CH), 55.51 (C-cyclopentyl), 120.09, 121.00, 126.03, 137.34 (C-phenyl), 140.09, 142.63, 149.46, 151.58, 152.07 (C-purine). MS (ESI+) *m/z*: 322.5 (100%) (M+H). HRMS (m/z) [M+H]^+^ Calculated: 322.2032, Found: 322.2026.

##### 4.2.3.2. 9-Cyclopentyl-*N*-(4-morpholinophenyl)-9*H*-purin-6-amine (9)

The above procedure was followed with 4-morpholinoaniline to yield **9** (0.11 g, 44% yield). Mp = 206–208 °C. ^1^H NMR (DMSO-*d_6_*) δ 1.66–1.71 (m, 2H), 1.85–1.90 (m, 2H), 1.96–2.05 (m, 2H), 2.11–2.19 (m, 2H), 3.03 (t, 4H), 3.72 (t, 4H), 4.84–4.88 (m, 1H), 6.89 (d, 2H, *J_o_* = 8.8 Hz), 7.73 (d, 2H, *J_o_* = 8.8 Hz), 8.29 (s, 1H), 8.31 (s, 1H), 9.58 (s, 1H). ^13^C NMR (DMSO-*d_6_*) δ 23.57, 31.91 (C-cyclopentyl), 49.08 (C-morpholine), 55.46 (C-cyclopentyl), 66.11, (C-morpholine), 115.28, 119.94, 122.08, 131.87 (C-phenyl), 139.82, 146.92, 149.29, 151.66, 152.11 (C-purine), 126.50, 127.10, 128.11, 138.02 (C-phenyl), 139.23, 140.20, 151.46, 152.03, 154.32 (C-purine). MS (ESI+) *m/z*: 365.5 (100%) (M+H). HRMS (m/z) [M+H]^+^ Calculated: 365.2090, Found: 365.2079.

All NMR, mass, and HRMS spectra are provided in the Supplementary information.

### 4.3. Cell cultures

Cells were maintained under standard incubator conditions at 37 °C and 5% CO_2_. Ovarian cancer cell lines OVCAR-3, OVSAHO, and KURAMOCHI were cultured in RPMI-1640 medium supplemented with 10% fetal bovine serum (FBS) and 1% penicillin-streptomycin.

### 4.4. In vitro cytotoxicity screening using the SRB assay

To assess compound cytotoxicity, cells were seeded into 96-well plates at a density of 5000 cells per well. After a 24-h incubation period to allow for cell attachment, treatments were initiated with increasing concentrations of the test compounds, ranging from 0.1 to 50 μM. The cells were then incubated for 72 h. Following treatment, cells were fixed with 10% trichloroacetic acid for 1 h at 4 °C, thoroughly washed with distilled water, and air-dried. The next day, fixed cells were stained with 0.4% SRB solution prepared in 1% acetic acid for 30 min at room temperature in the dark. Excess dye was removed with several washes of 1% acetic acid. After air-drying, the protein-bound dye was solubilized in 10 mM Tris-base and absorbance was measured at 515 nm. DMSO was used as a negative control (0.005% v/v for the highest dose and serially diluted thereafter). All experiments were conducted in triplicate and repeated on three independent plates.

### 4.5. Flow cytometry for cell-cycle analysis

Cells (1 × 10^5^ cells/well) were seeded in 6-well plates and treated with 10 μM of the selected test compounds or DMSO as a control (0.001% v/v) for 72 h. After treatment, cells were washed with cold Dulbecco’s phosphate-buffered saline (DPBS) and fixed by gently adding 70% ethanol while applying low-speed vortexing. Following centrifugation to remove the fixative, the cell pellets were resuspended in a staining solution containing 50 μg/mL PI, 0.1 mg/mL RNase A, and 0.05% Triton X-100 in cold 1X DPBS. Samples were incubated at 37 °C for 40 min, washed three times with DPBS, and analyzed using a CytoFLEX flow cytometer (Beckman Coulter, Brea, CA, USA). Initial reads obtained using FSC-A/SSC-A and doublet cells in the read were filtered with the FSC-A/FSC-H table. The selected P2 gate was then subjected to the FSC-A/PE channel and histograms were obtained. Peaks were divided into four sections with horizontal gating, according to n – 2n numbers of cells. The described gates were taken in the DMSO control sample and were applied to treatment samples to ensure data accuracy.

### 4.6. Western blotting

Cells were plated in 15-cm dishes with 5 × 10^6^ cells/dish and treated with the IC_50_ or IC_75_ concentrations of test compounds for 48 h. Cell lysis was performed with RIPA buffer containing protease and phosphatase inhibitors. Lysates were incubated on ice, vortexed, and centrifuged, and protein concentrations were determined via BCA assay. Equal protein amounts (20 μg) were denatured, loaded onto gradient concentrations of 4%–15% SDS-PAGE gels, and transferred to PVDF membranes. Membranes were blocked overnight, probed with primary antibodies against PARP (116 kDa when full length and 89 kDa when cleaved), BCL-2 (26 kDa), cyclin E2 (50 kDa), and cleaved caspase-3 (17 kDa) (1:1000, 1:250, 1:1000, and 1:250 dilutions respectively), followed by HRP-conjugated secondary antibodies. The housekeeping protein α-tubulin (1:1000) was used as a loading control. Chemiluminescent detection was performed with electrochemiluminescence with exposure times ranging from 30 s to 2 min and bands were visualized using a LI-COR system (LI-COR Biotechnology, Lincoln, NE, USA). Band intensity was quantified using open-source ImageJ software and statistical analysis was performed with GraphPad Prism (GraphPad Software, La Jolla, CA, USA). Western blots were repeated with replicates and the best visual representations were added to the results.

### 4.7. Statistical analysis

All statistical evaluations were conducted using GraphPad Prism software. Data were primarily analyzed using unpaired two-tailed t-tests. Where applicable, one-way ANOVA was employed to assess statistical differences between treated and control groups. Statistical significance was denoted as follows: **p < 0.05, ***p < 0.01, and ****p < 0.001.

## Supplementary Data

Figure S1 ^1^H NMR spectrum of Compound 2.

Figure S2 ^13^C NMR spectrum of Compound 2.

Figure S3 Mass spectrum of Compound 2.

Figure S4 ^1^H NMR spectrum of Compound 3.

Figure S5 ^13^C NMR spectrum of Compound 3.

Figure S6 Mass spectrum of Compound 3.

Figure S7 HRMS spectrum of Compound 3.

Figure S8 ^1^H NMR spectrum of Compound 4.

Figure S9 ^13^C NMR spectrum of Compound 4.

Figure S10 Mass spectrum of Compound 4.

Figure S11 HRMS spectrum of Compound 4.

Figure S12 ^1^H NMR spectrum of Compound 5.

Figure S13 ^13^C NMR spectrum of Compound 5.

Figure S14 Mass spectrum of Compound 5.

Figure S15 HRMS spectrum of Compound 5.

Figure S16 ^1^H NMR spectrum of Compound 6.

Figure S17 ^13^C NMR spectrum of Compound 6.

Figure S18 Mass spectrum of Compound 6.

Figure S19 HRMS spectrum of Compound 6.

Figure S20 ^1^H NMR spectrum of Compound 7.

Figure S21 ^13^C NMR spectrum of Compound 7.

Figure S22 Mass spectrum of Compound 7.

Figure S23 ^1^H NMR spectrum of Compound 8.

Figure S24 ^13^C NMR spectrum of Compound 8..

Figure S25 Mass spectrum of Compound 8.

Figure S26. HRMS spectrum of Compound 8.

Figure S27 ^1^H NMR spectrum of Compound 9.

Figure S28 ^13^C NMR spectrum of Compound 9.

Figure S29 Mass spectrum of Compound 9.

Figure S30 HRMS spectrum of Compound 9.

## Figures and Tables

**Table:** IC_50_ values of the compounds derived from NCI SRB assay results. Values represent the mean IC_50_ (μM) ± standard deviation from three independent biological replicates (n = 3; R^2^ > 0.8).

Compound	HGSOC cell lines, IC_50_ (μM)^a^		

	OVSAHO	OVCAR-3	KURAMOCHI
**3**	>50 μM	>50 μM	>50 μM
**4**	>50 μM	>50 μM	>50 μM
**5**	>50 μM	13.6 μM ± 3.3	>50 μM
**6**	>50 μM	15.7 μM ± 2.3	>50 μM
**7**	>50 μM	21.75 μM ± 1.6	41.86 μM ± 2.6
**8**	11.3 μM ± 1.6	7.76 μM ± 1.2	48.1 μM ± 7.9
**9**	>50 μM	>50 μM	>50 μM
**Carboplatin**	55.9 μM ± 1.3	20.9 μM ± 2.2	32 μM ± 1.3
**Olaparib**	89.3 μM ± 10.7	75.8 μM ± 6.1	84.5 μM ± 11.4
