# Supplementary figures and images for: Biological evaluation of novel 6,9-disubstituted purine analogues in high-grade serous ovarian cancer cell lines
Source: Turk J Biol. 2025 Dec 18;50(1):29–36. doi: 10.55730/1300-0152.2788 (PMC12978765; doi:10.55730/1300-0152.2788)

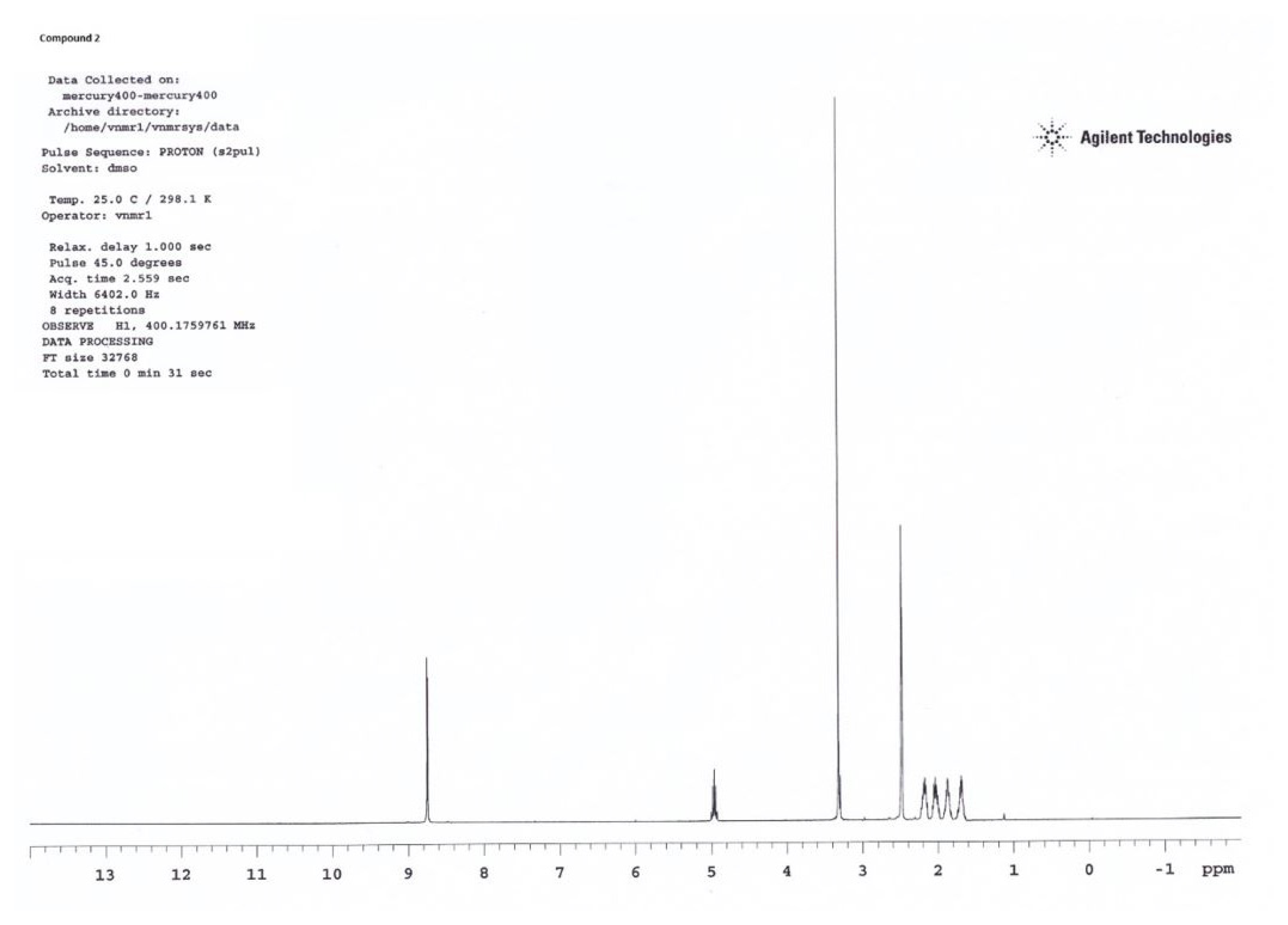

Supplement: Figure S1 — 1H NMR spectrum of Compound 2. [file tjb-50-01-29s1.tif]

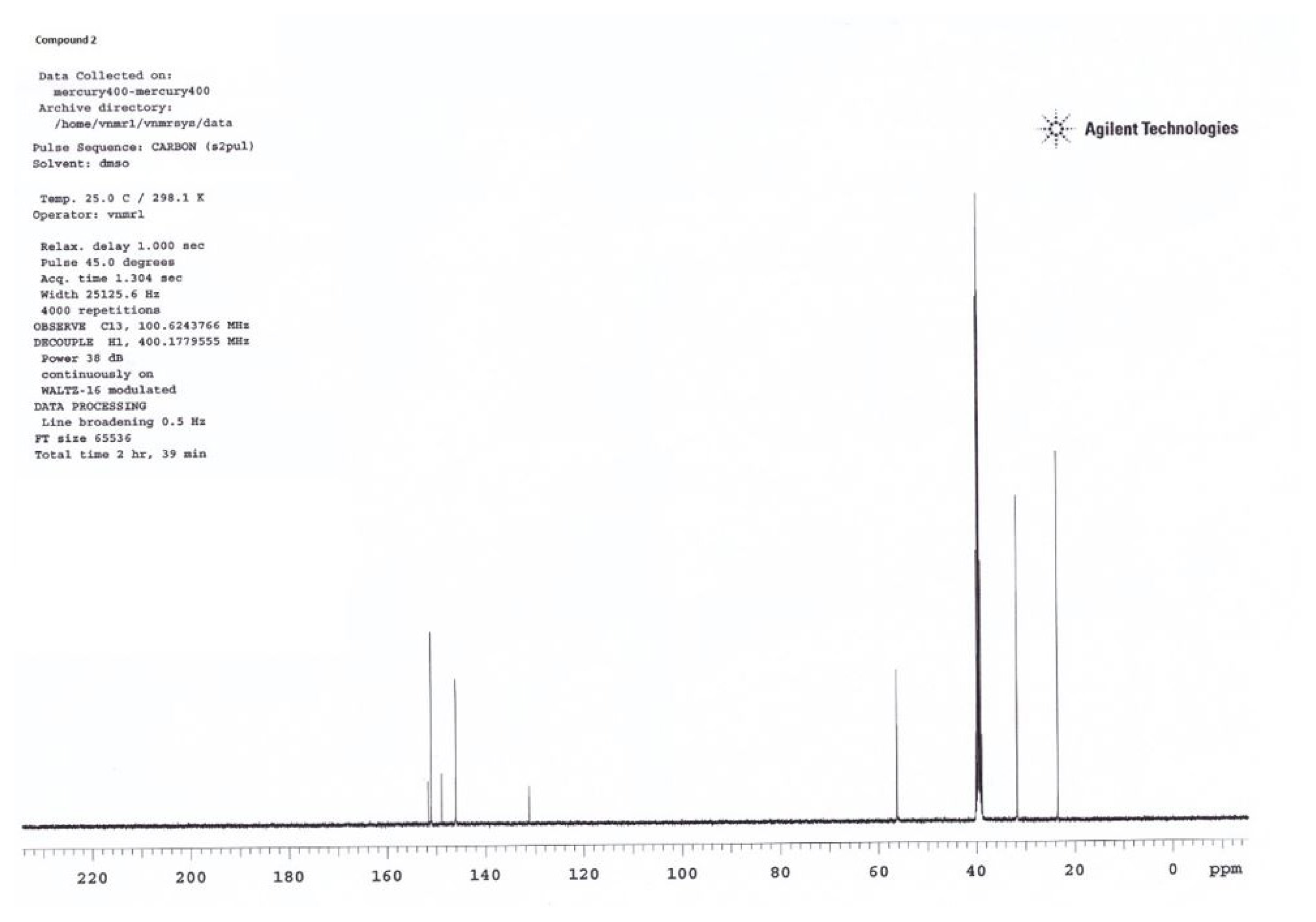

Supplement: Figure S2 — 13C NMR spectrum of Compound 2. [file tjb-50-01-29s2.tif]

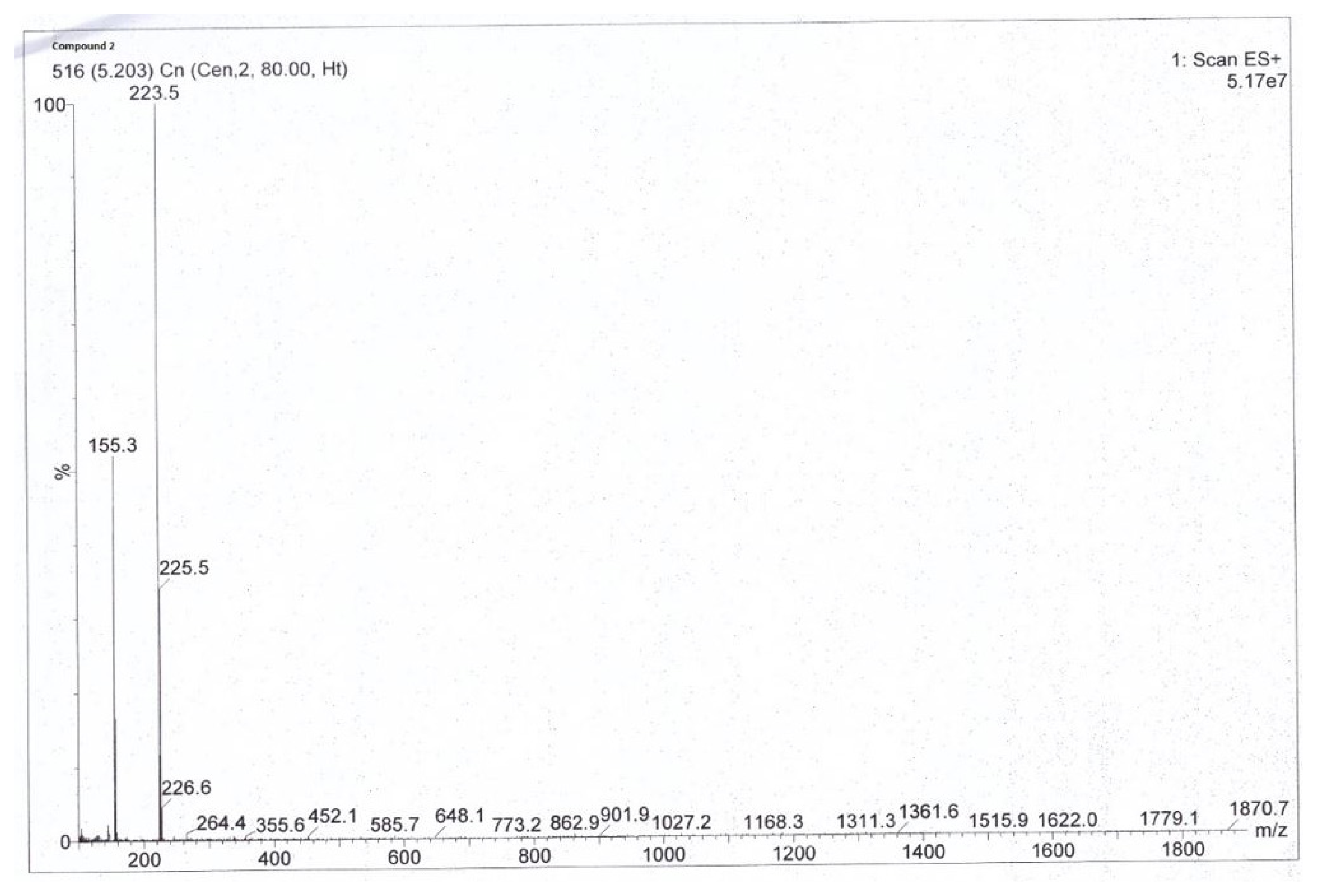

Supplement: Figure S3 — Mass spectrum of Compound 2. [file tjb-50-01-29s3.tif]

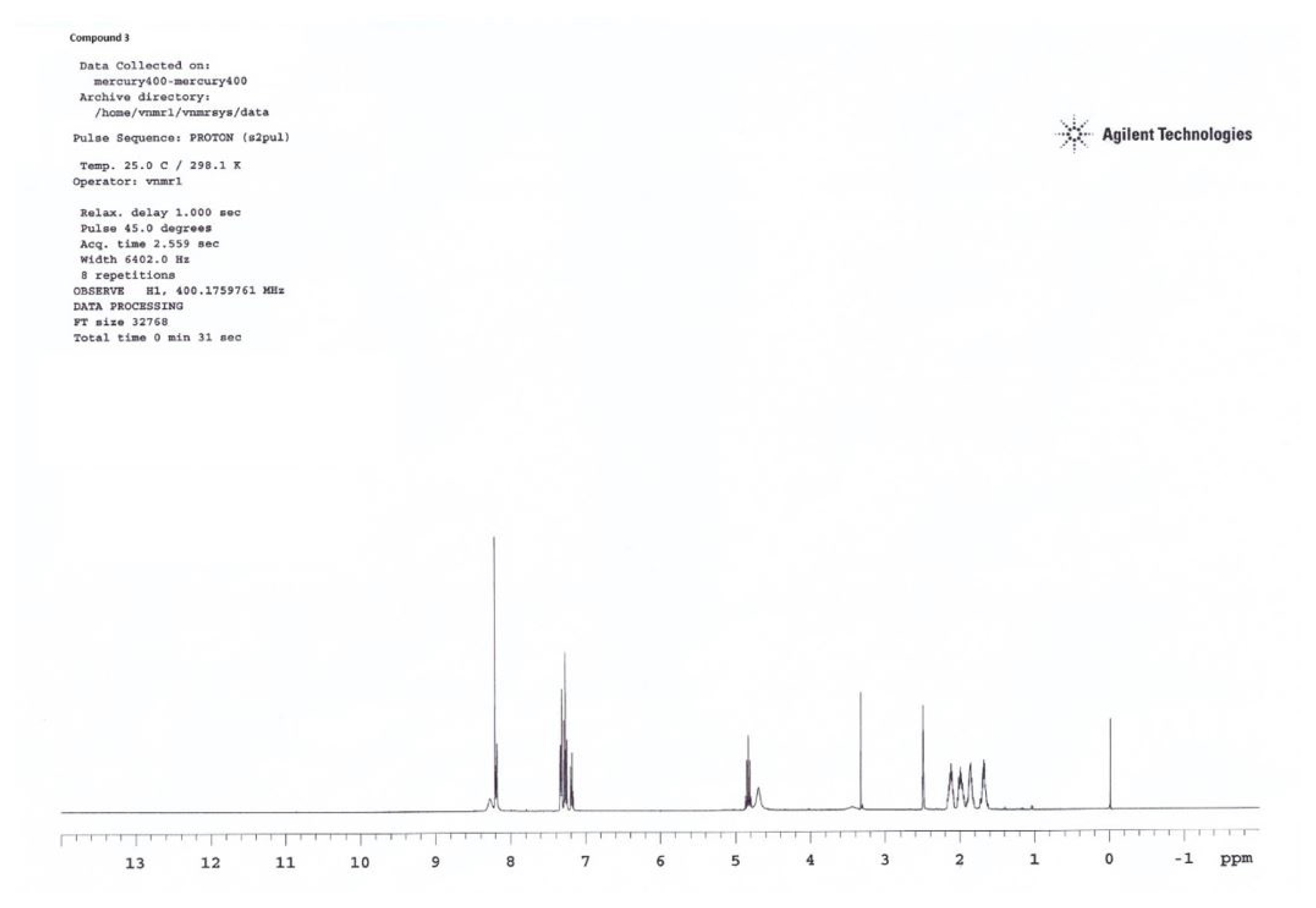

Supplement: Figure S4 — 1H NMR spectrum of Compound 3. [file tjb-50-01-29s4.tif]

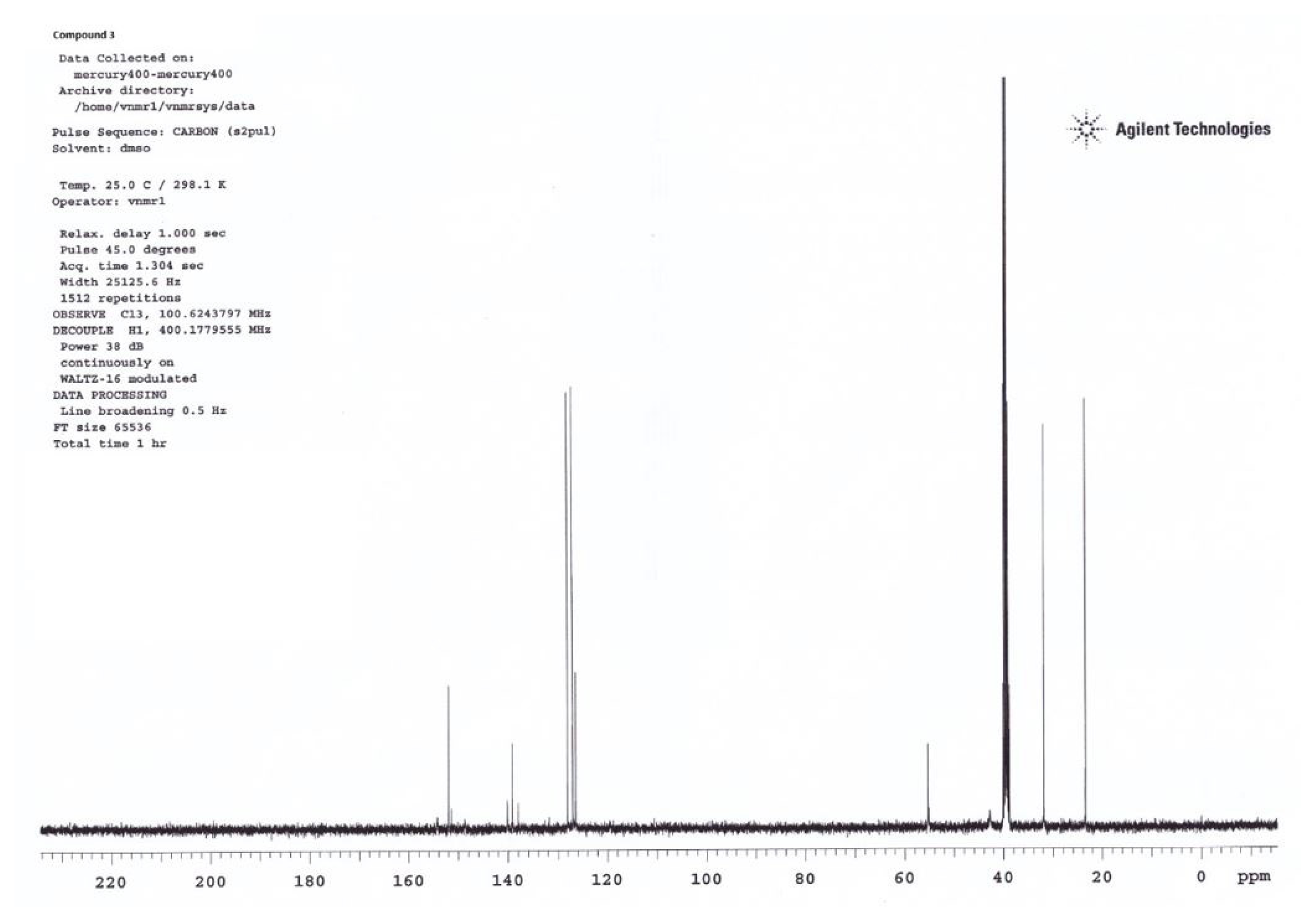

Supplement: Figure S5 — 13C NMR spectrum of Compound 3. [file tjb-50-01-29s5.tif]

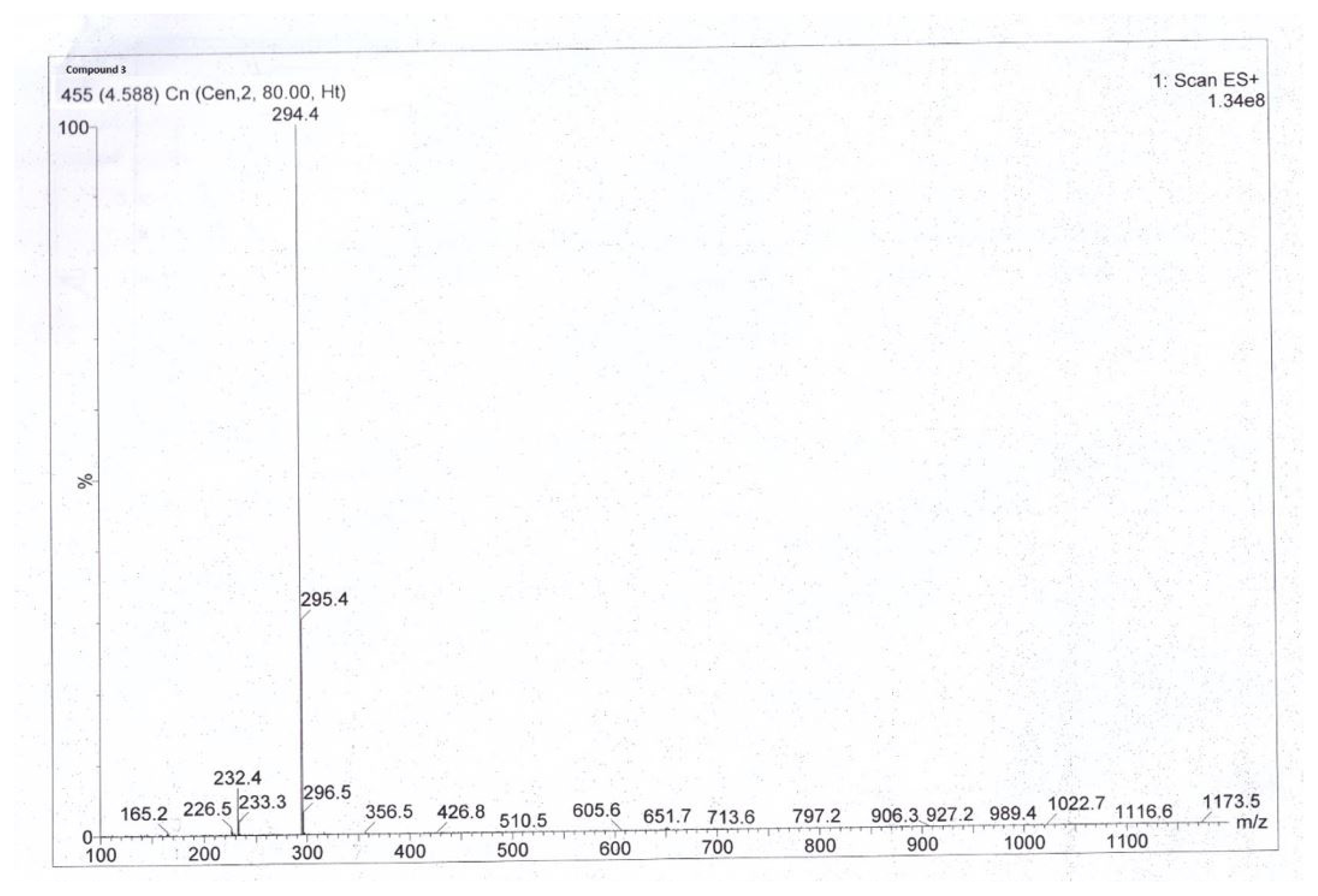

Supplement: Figure S6 — Mass spectrum of Compound 3. [file tjb-50-01-29s6.tif]

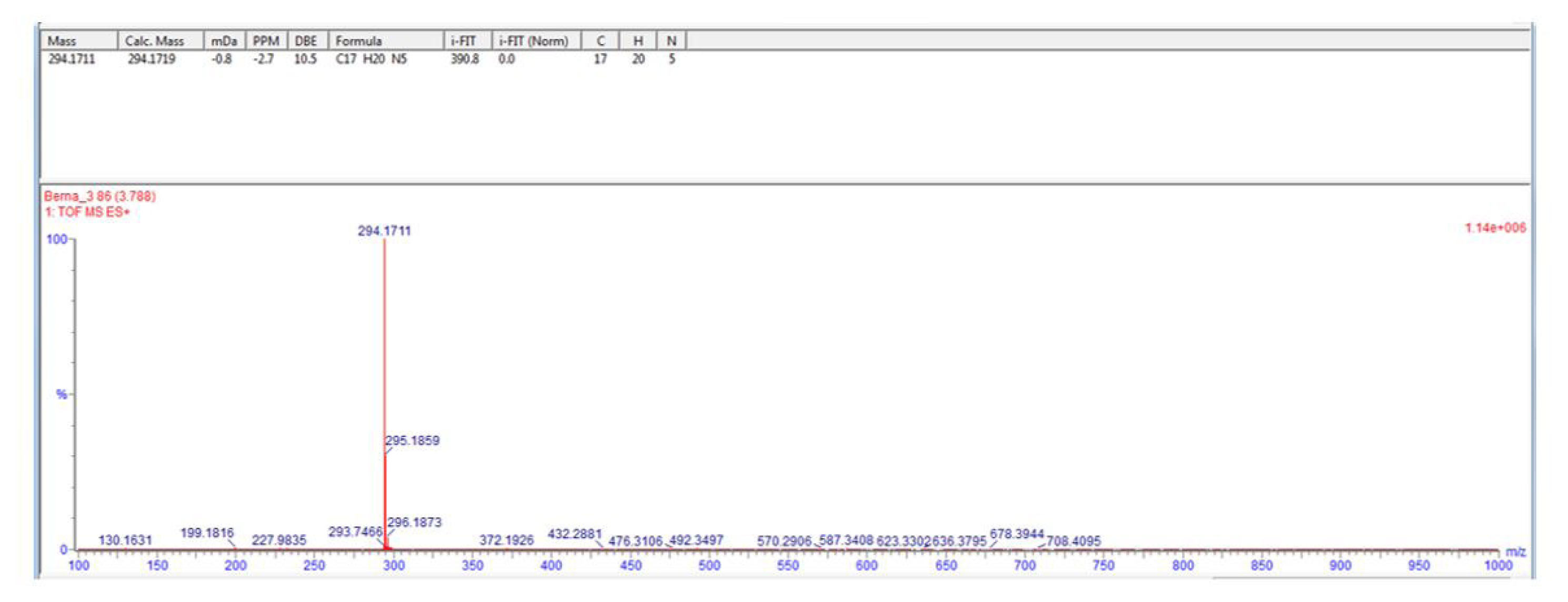

Supplement: Figure S7 — HRMS spectrum of Compound 3. [file tjb-50-01-29s7.tif]

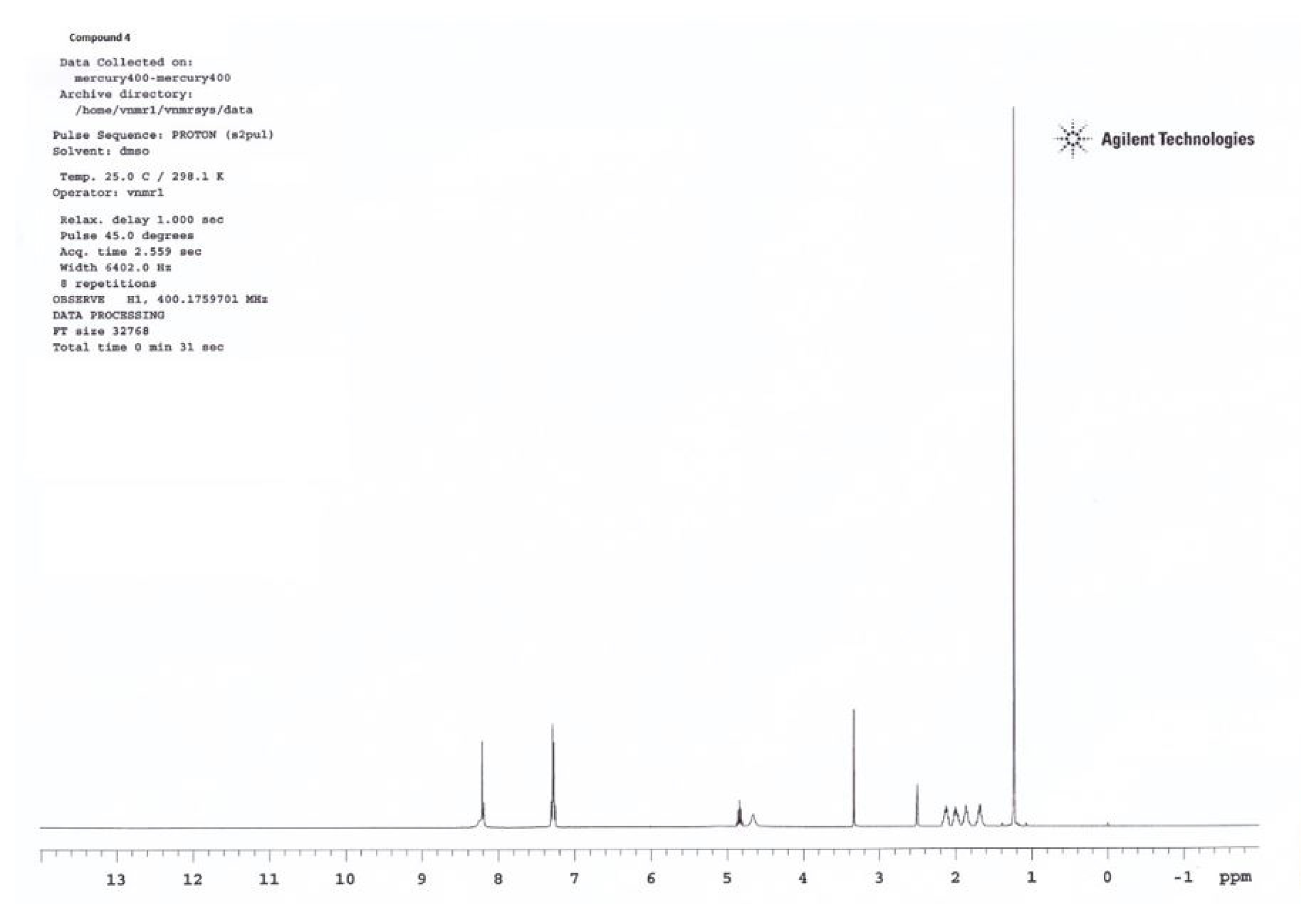

Supplement: Figure S8 — 1H NMR spectrum of Compound 4. [file tjb-50-01-29s8.tif]

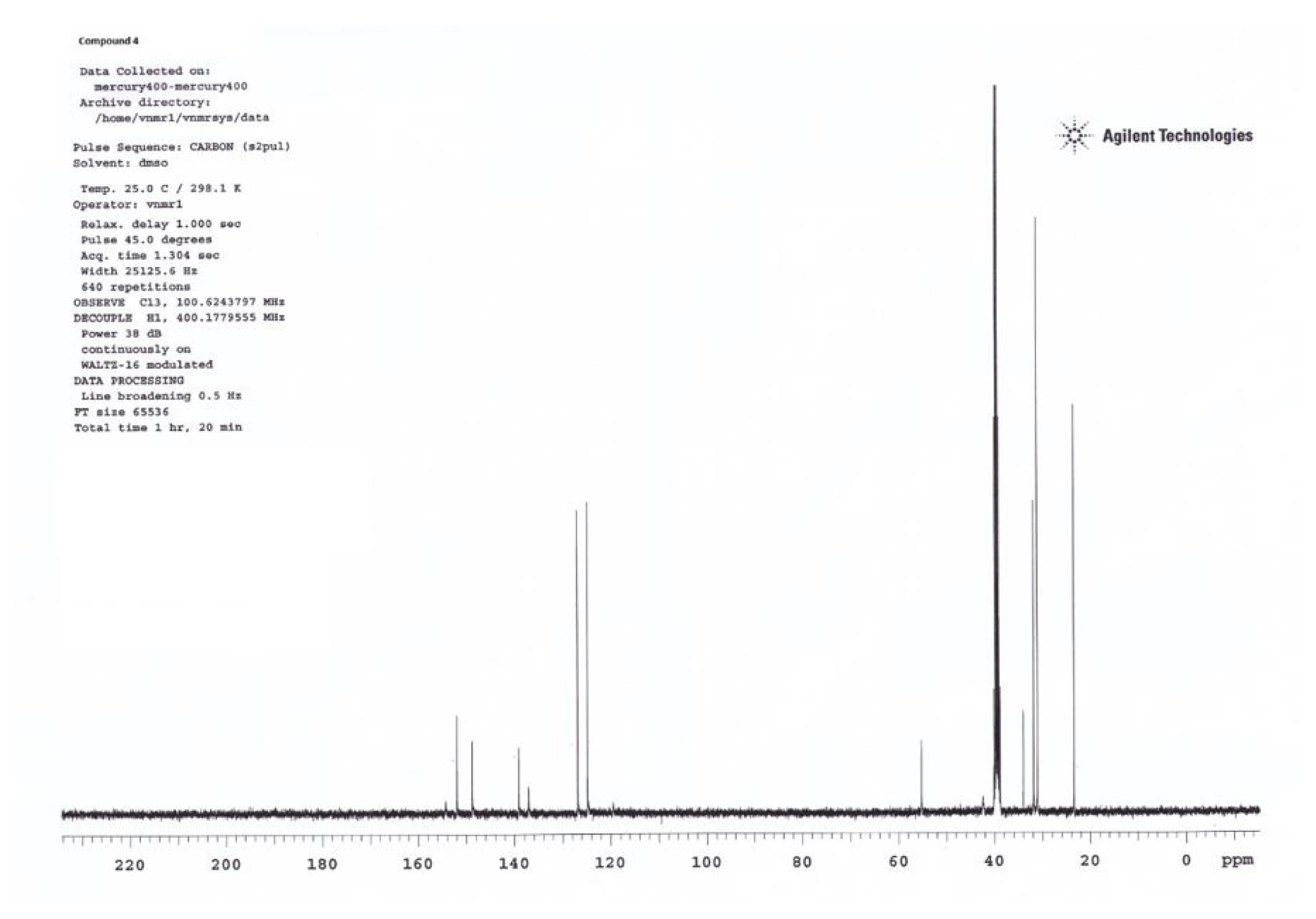

Supplement: Figure S9 — 13C NMR spectrum of Compound 4. [file tjb-50-01-29s9.tif]

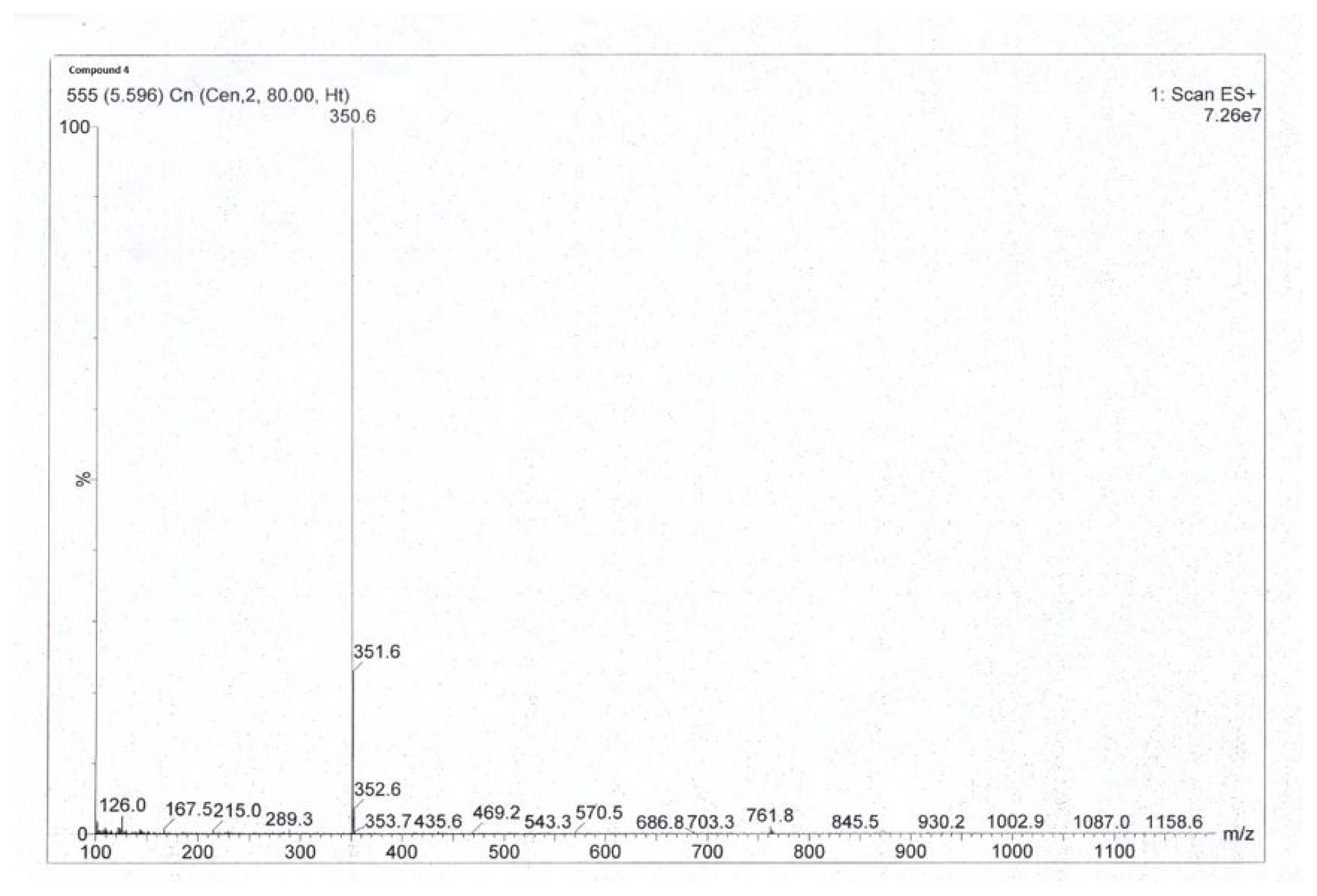

Supplement: Figure S10 — Mass spectrum of Compound 4. [file tjb-50-01-29s10.tif]

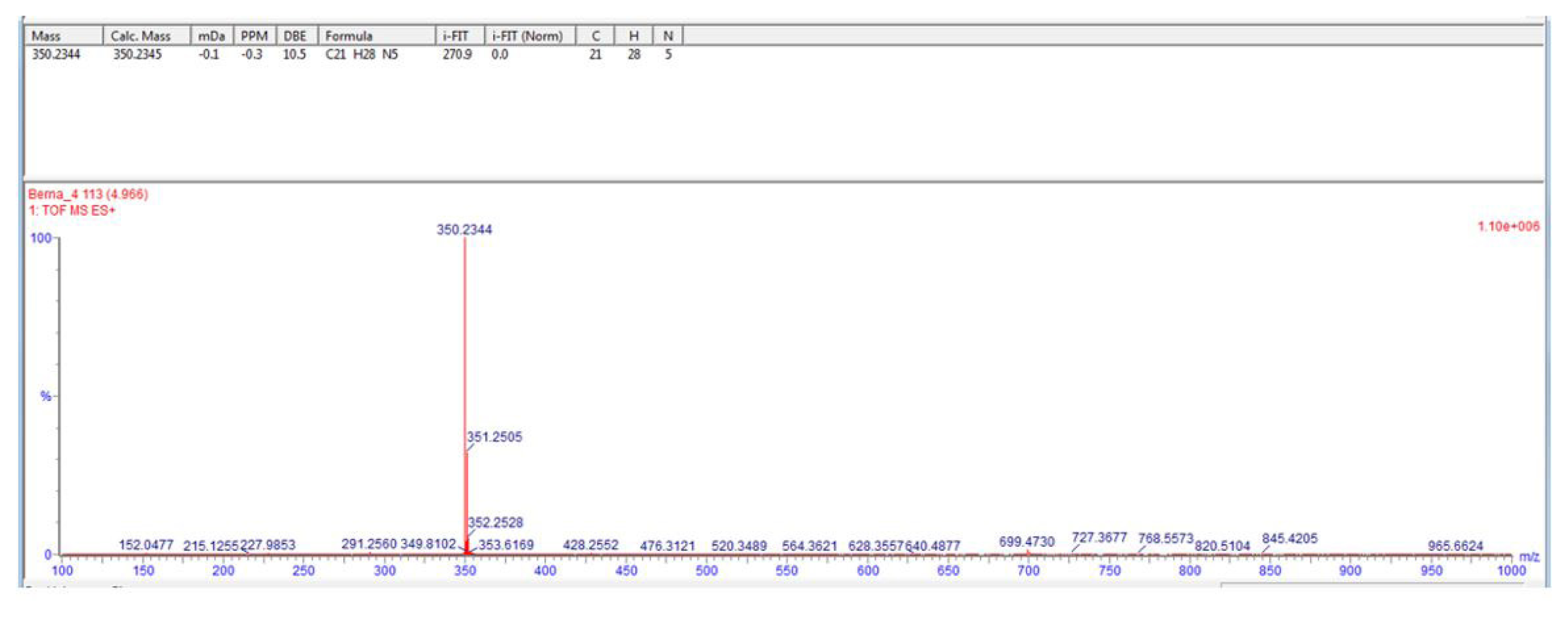

Supplement: Figure S11 — HRMS spectrum of Compound 4. [file tjb-50-01-29s11.tif]

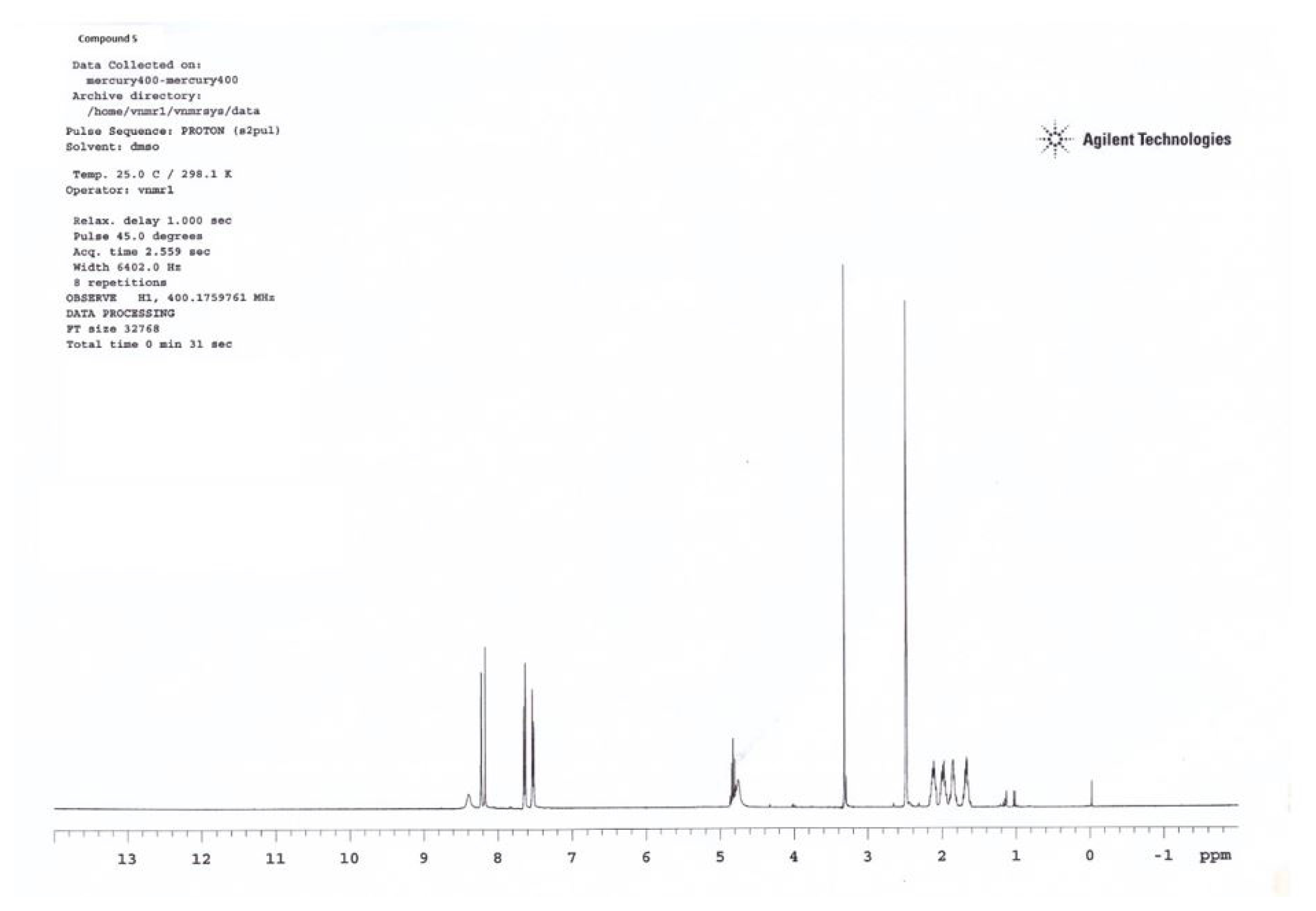

Supplement: Figure S12 — 1H NMR spectrum of Compound 5. [file tjb-50-01-29s12.tif]

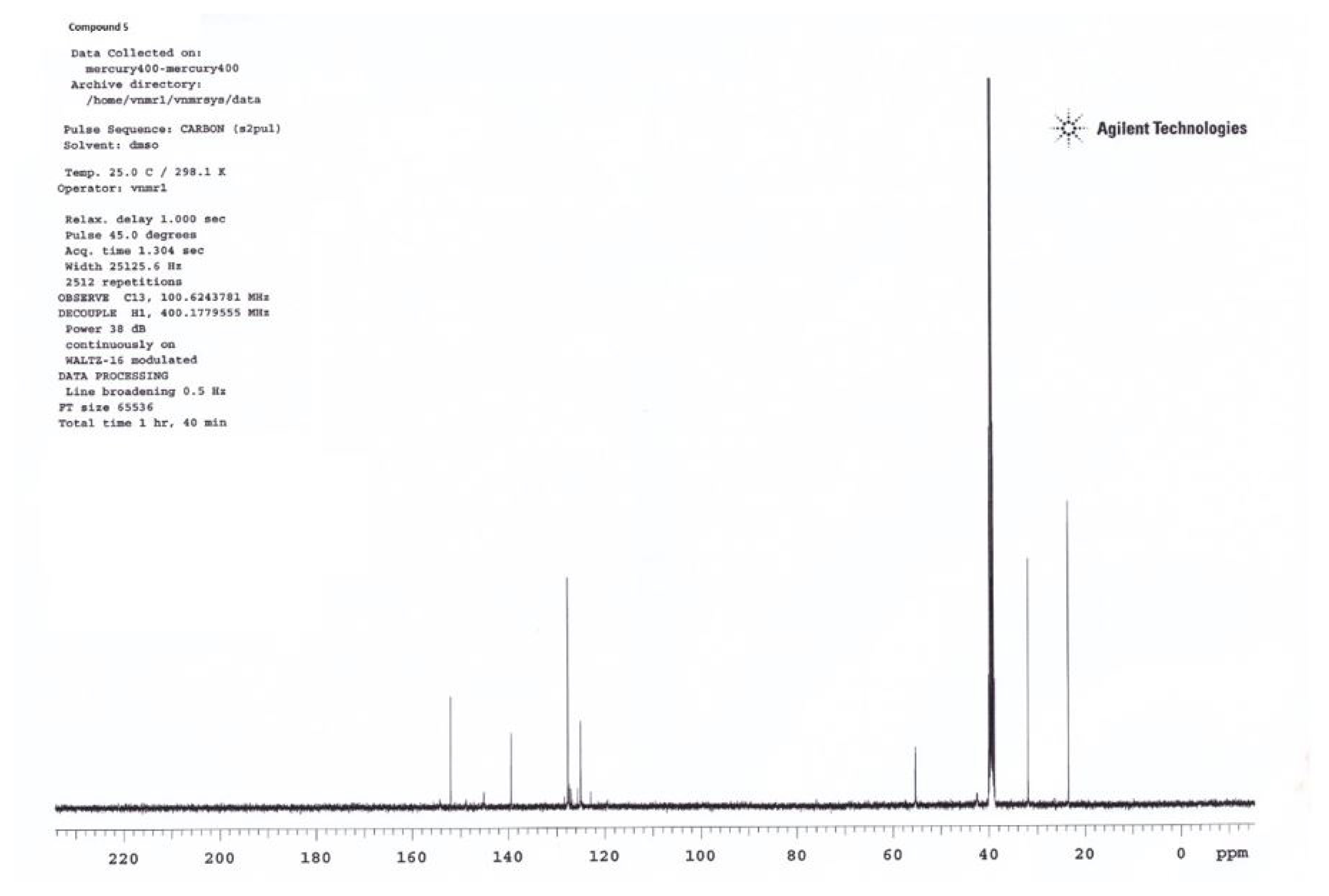

Supplement: Figure S13 — 13C NMR spectrum of Compound 5. [file tjb-50-01-29s13.tif]

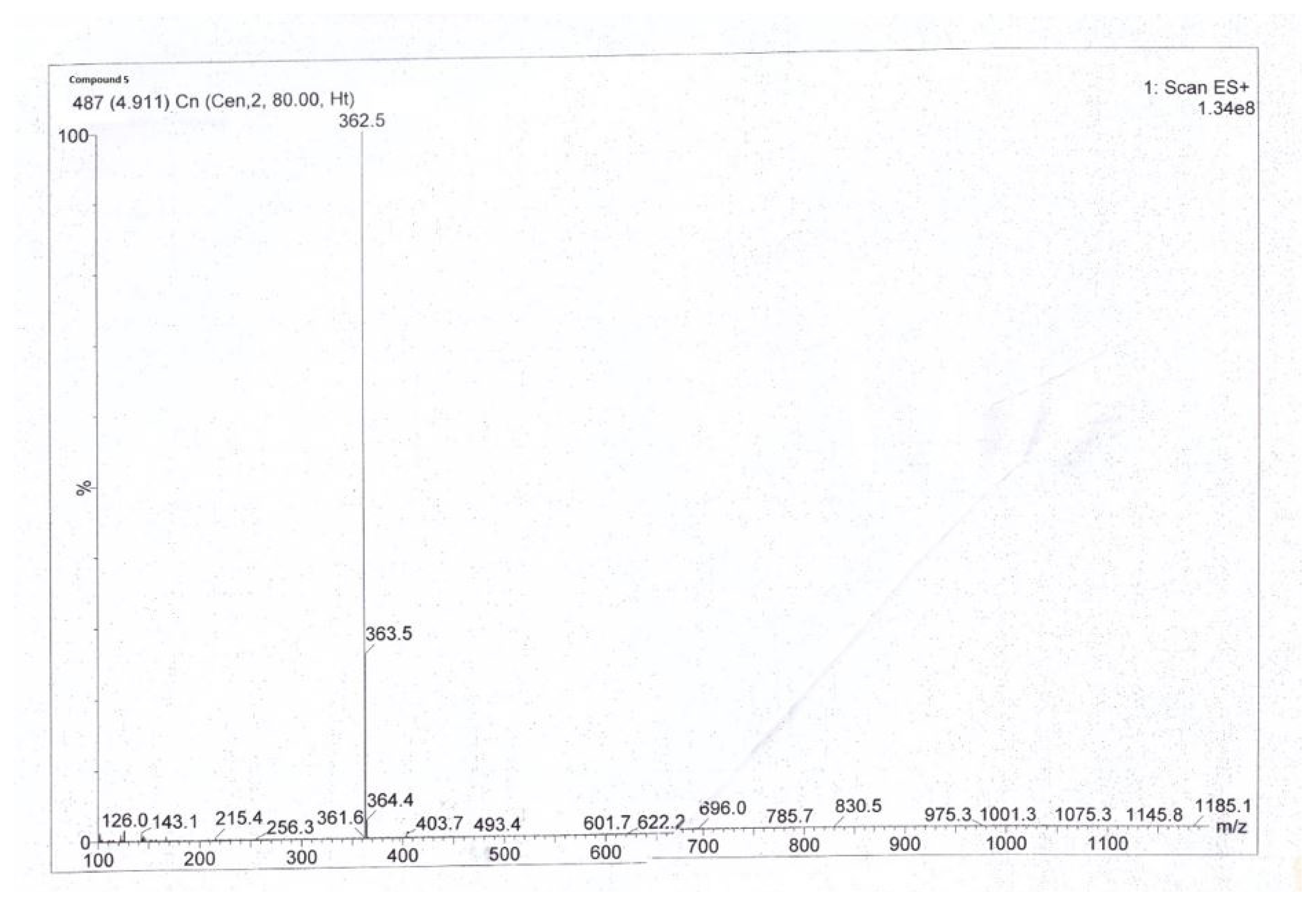

Supplement: Figure S14 — Mass spectrum of Compound 5. [file tjb-50-01-29s14.tif]

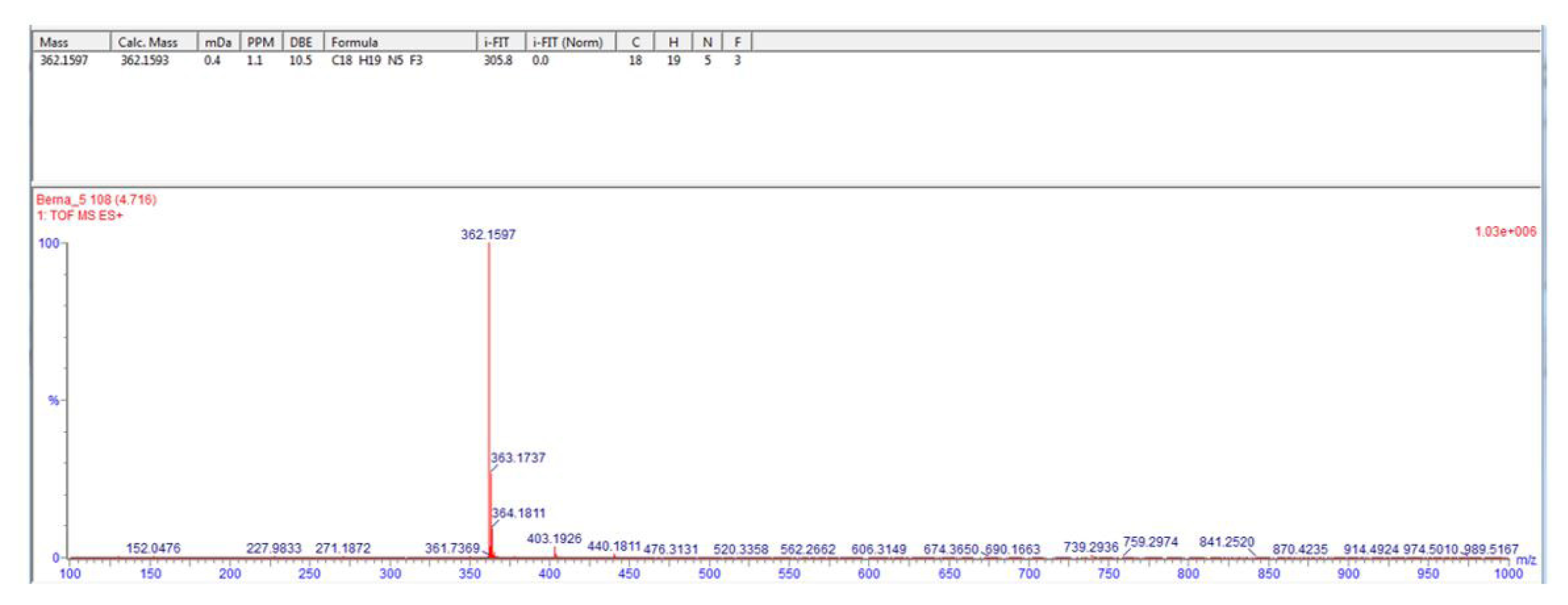

Supplement: Figure S15 — HRMS spectrum of Compound 5. [file tjb-50-01-29s15.tif]

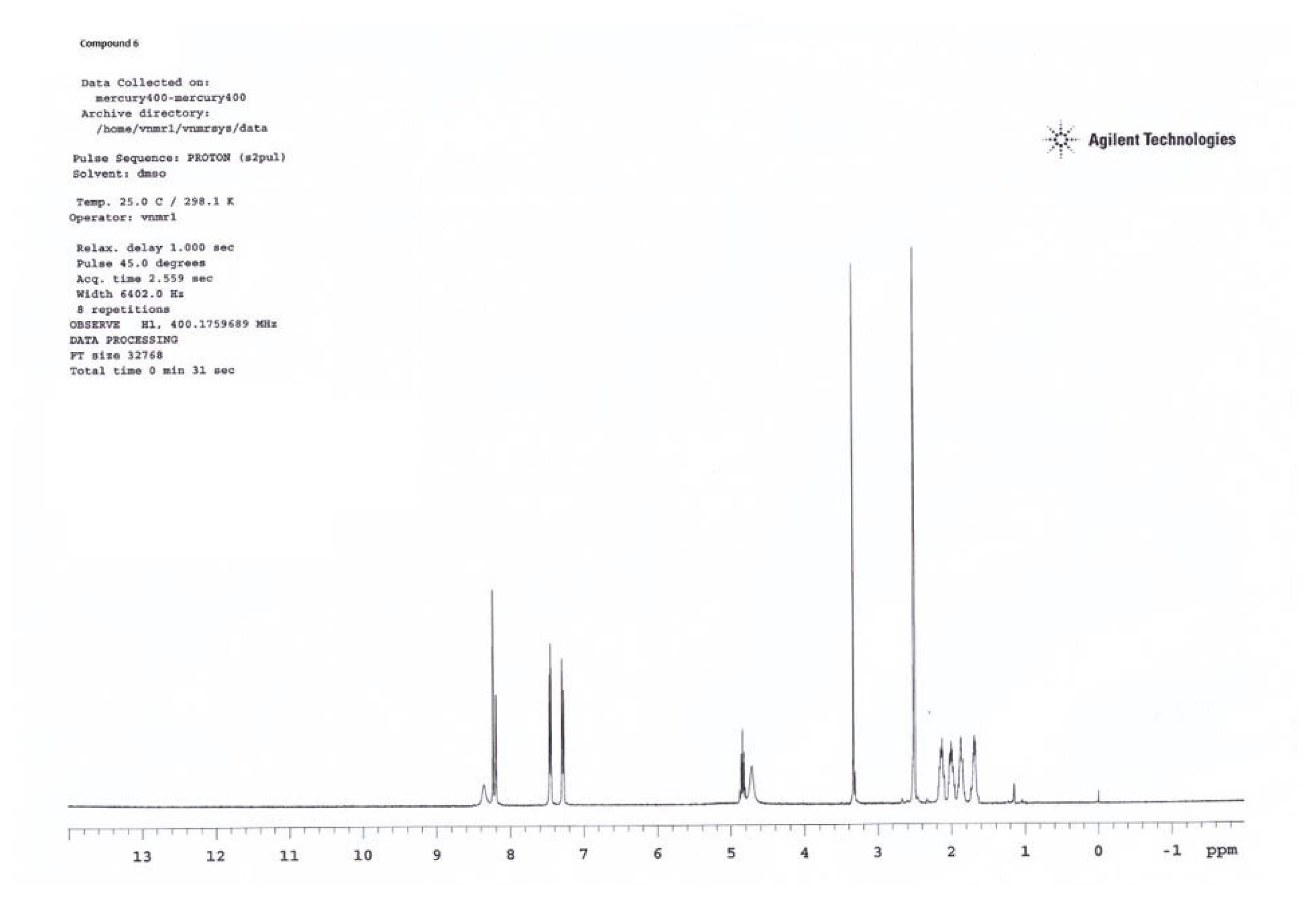

Supplement: Figure S16 — 1H NMR spectrum of Compound 6. [file tjb-50-01-29s16.tif]

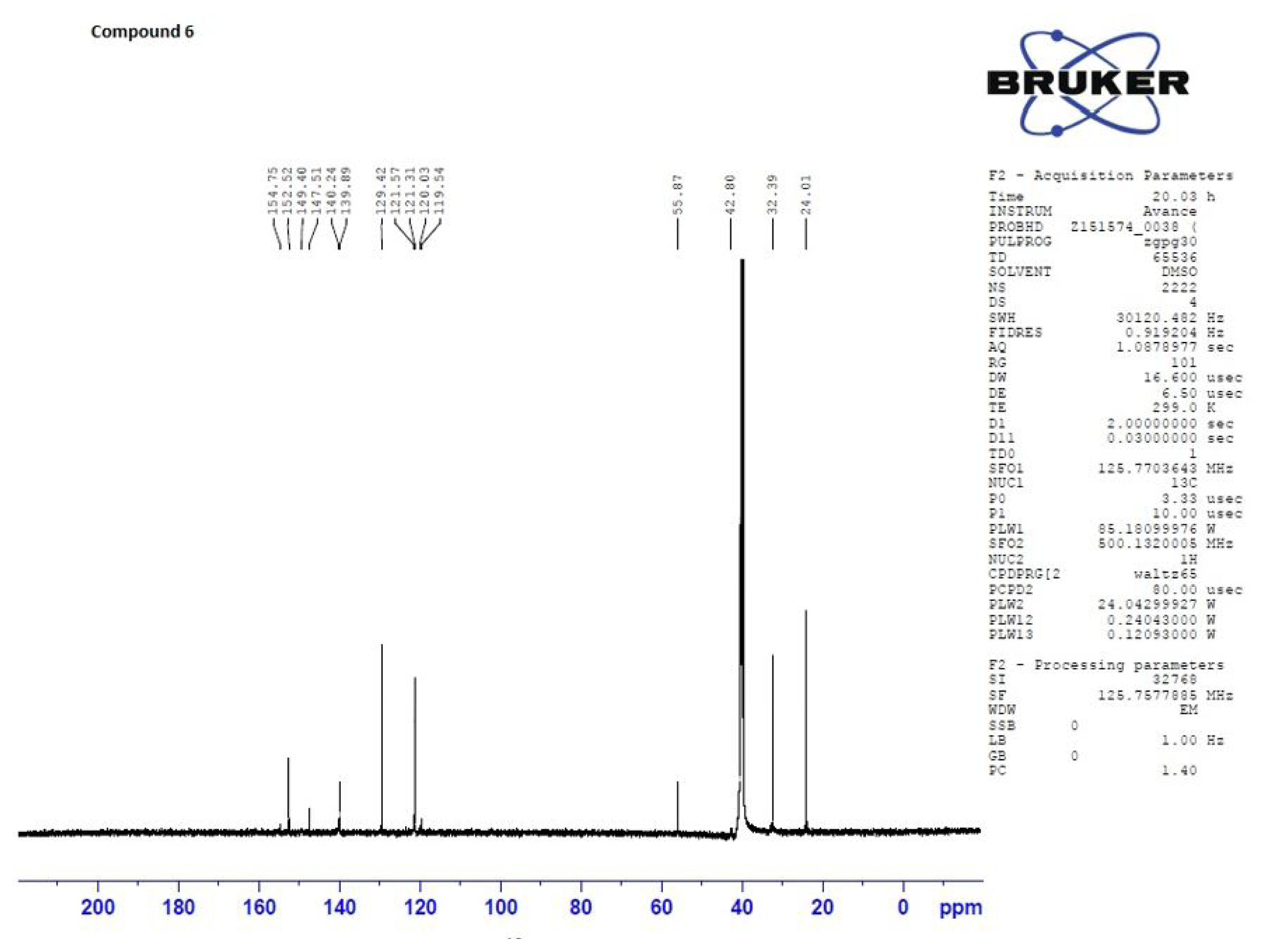

Supplement: Figure S17 — 13C NMR spectrum of Compound 6. [file tjb-50-01-29s17.tif]

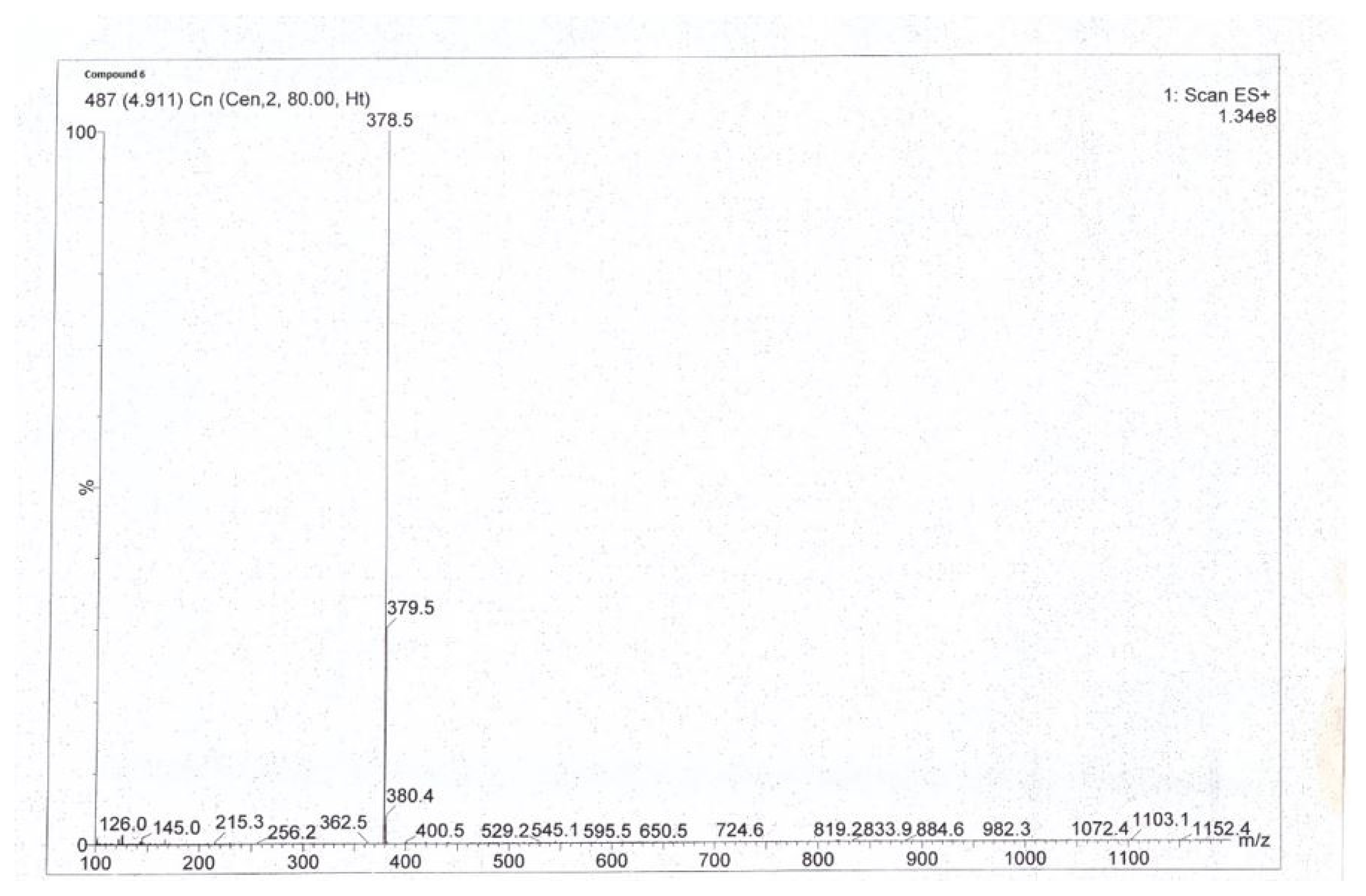

Supplement: Figure S18 — Mass spectrum of Compound 6. [file tjb-50-01-29s18.tif]

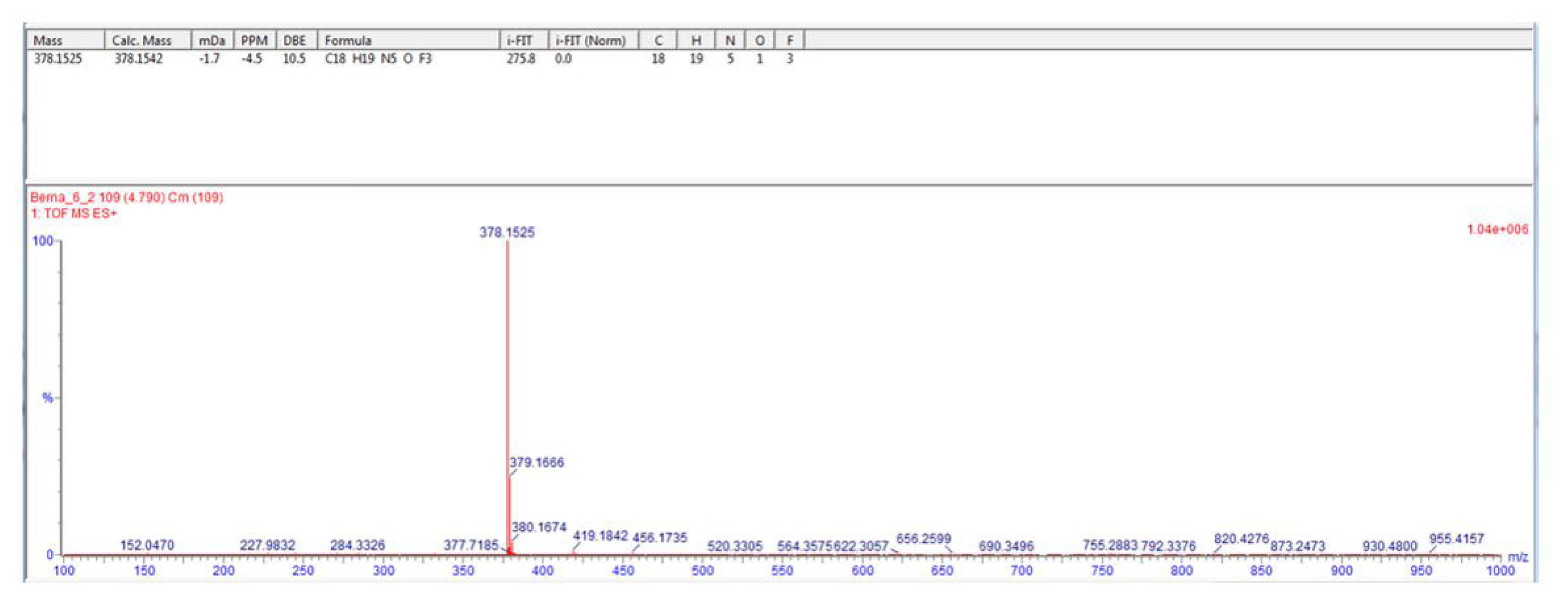

Supplement: Figure S19 — HRMS spectrum of Compound 6. [file tjb-50-01-29s19.tif]

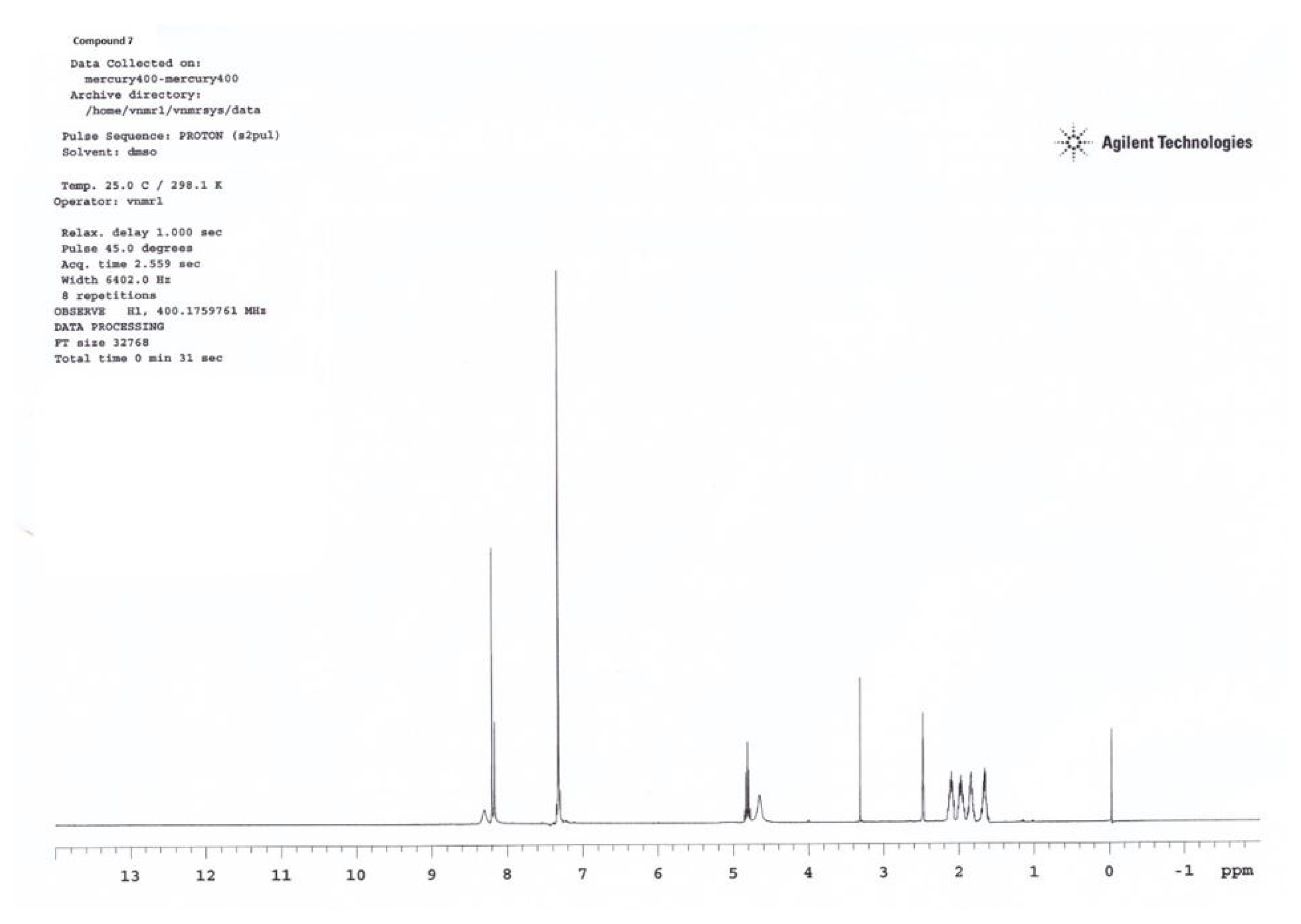

Supplement: Figure S20 — 1H NMR spectrum of Compound 7. [file tjb-50-01-29s20.tif]

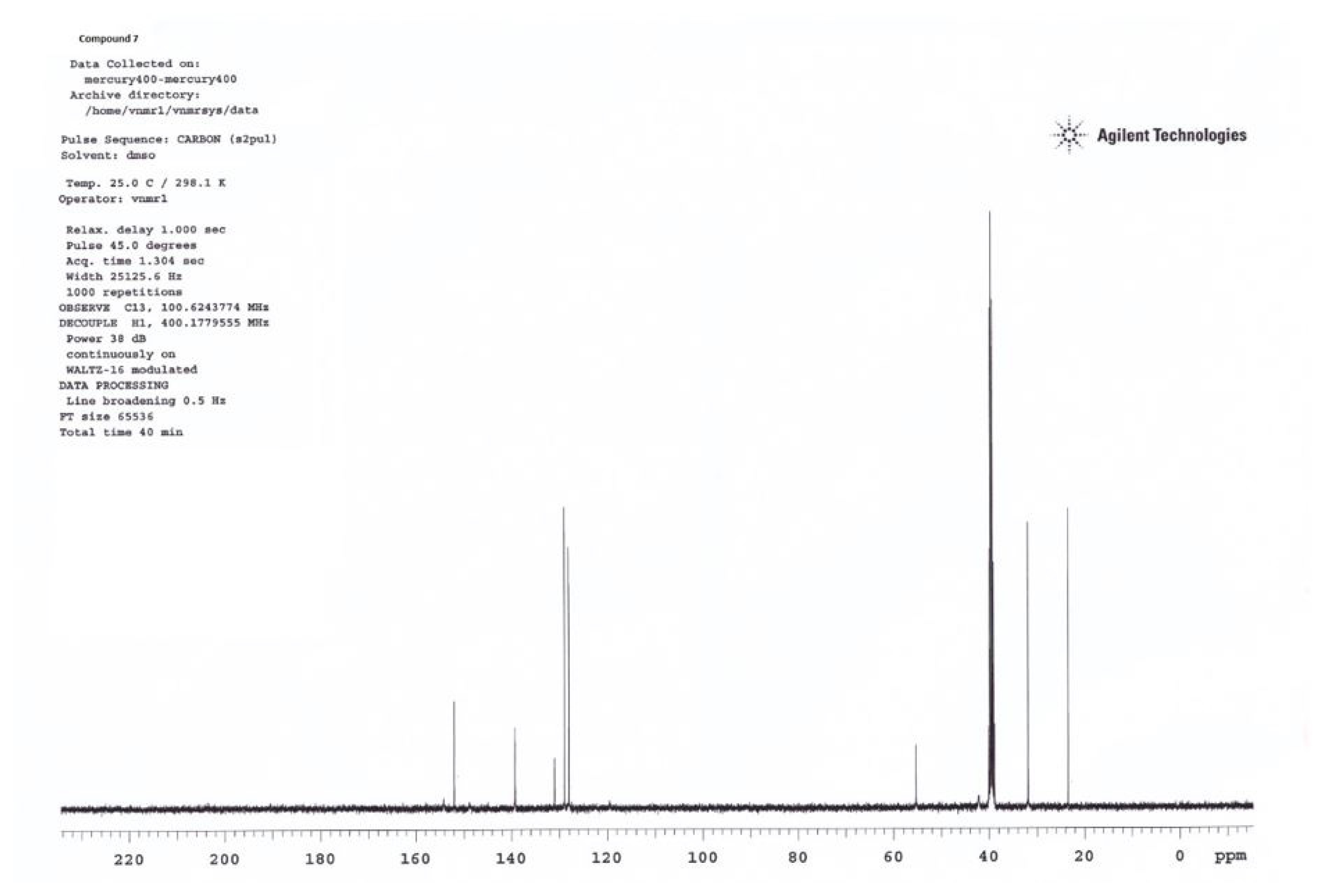

Supplement: Figure S21 — 13C NMR spectrum of Compound 7. [file tjb-50-01-29s21.tif]

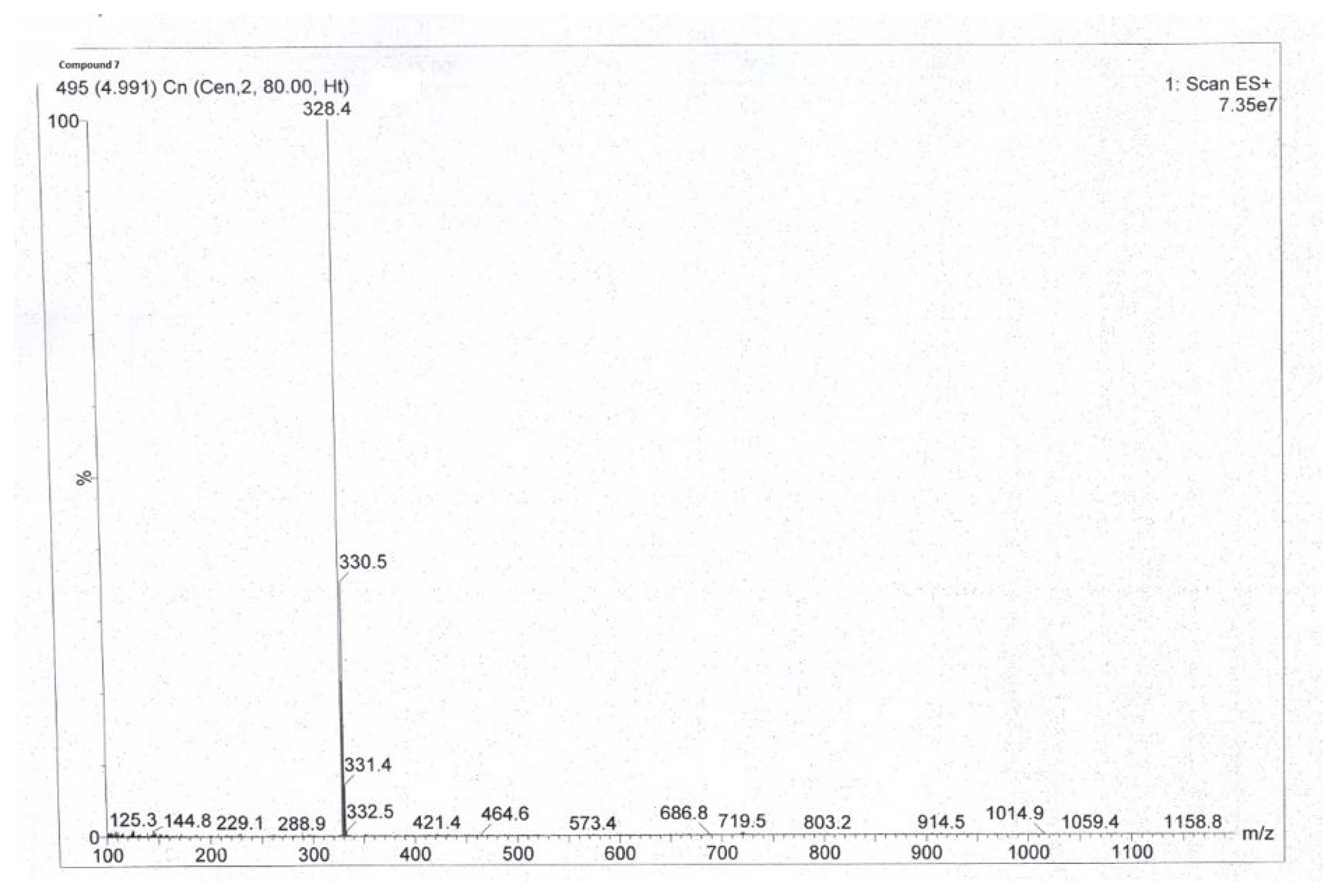

Supplement: Figure S22 — Mass spectrum of Compound 7. [file tjb-50-01-29s22.tif]

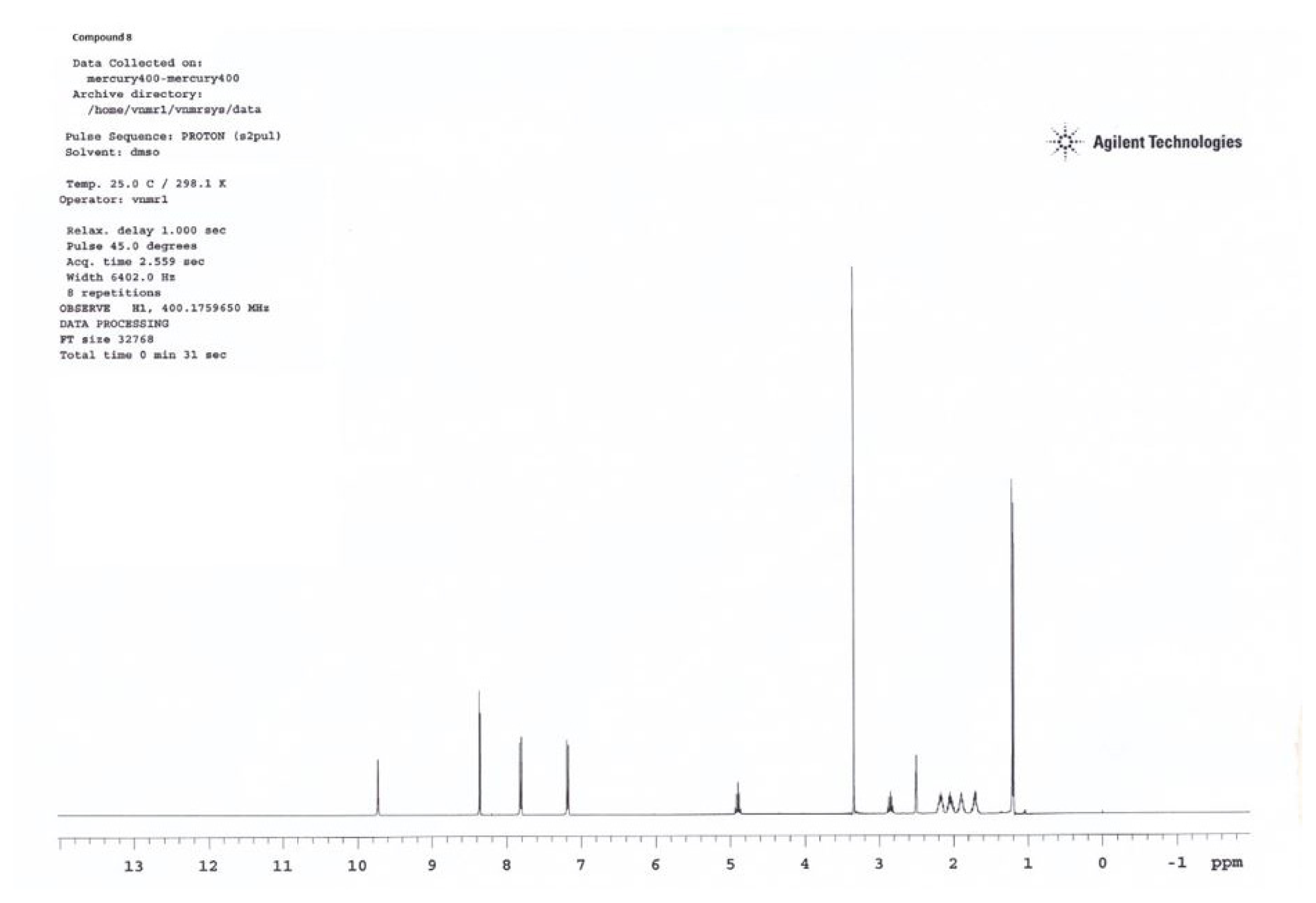

Supplement: Figure S23 — 1H NMR spectrum of Compound 8. [file tjb-50-01-29s23.tif]

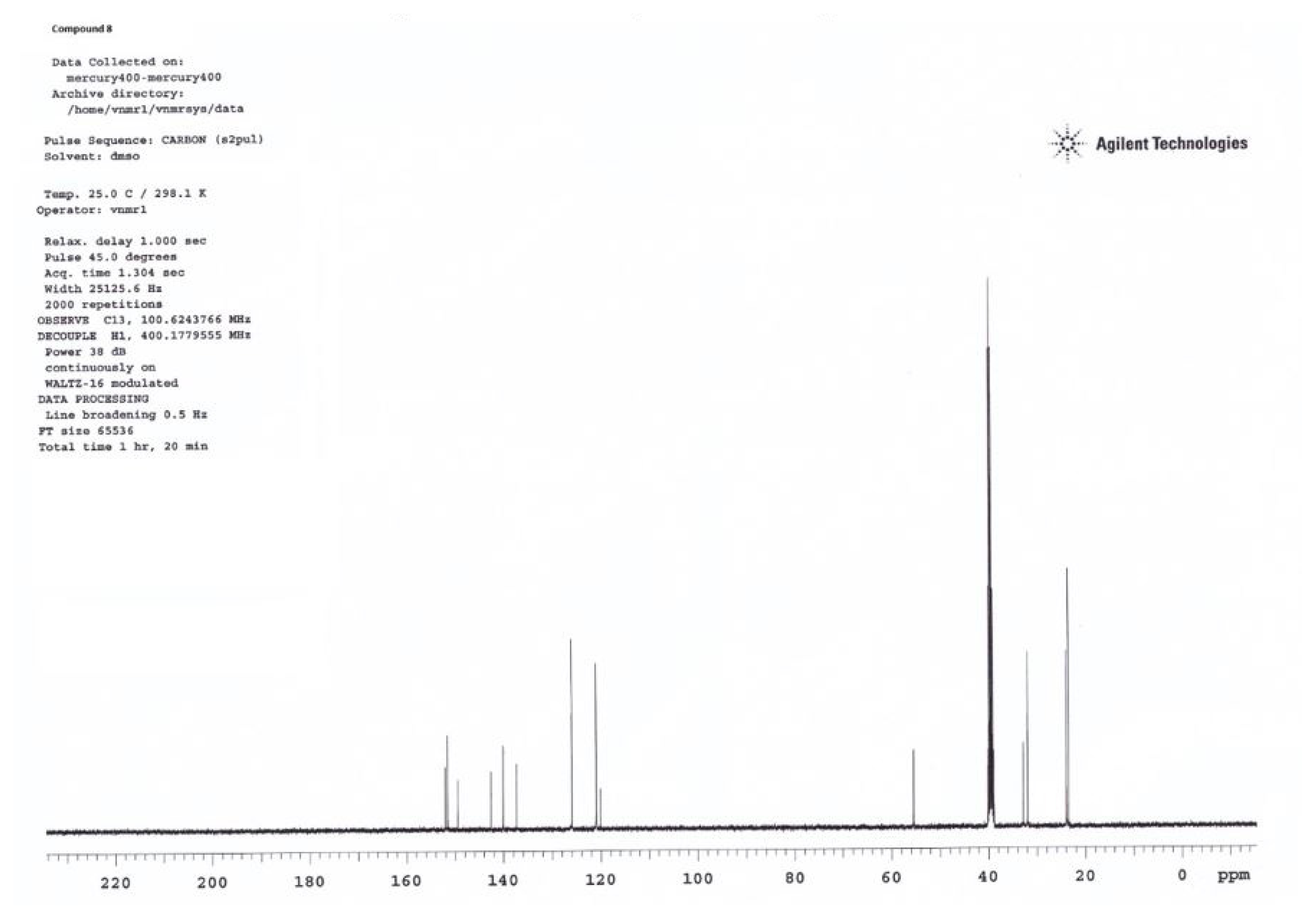

Supplement: Figure S24 — 13C NMR spectrum of Compound 8.. [file tjb-50-01-29s24.tif]

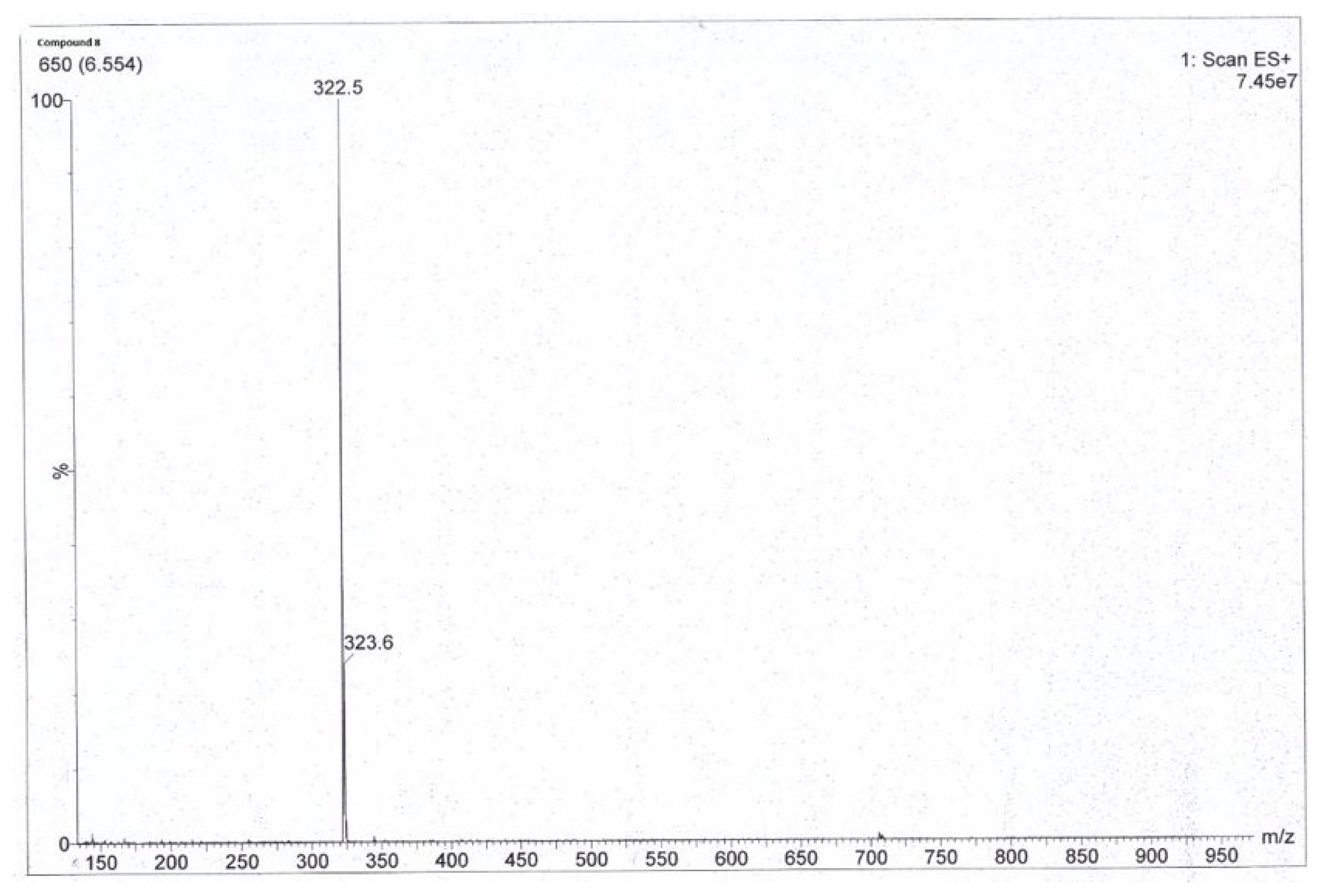

Supplement: Figure S25 — Mass spectrum of Compound 8. [file tjb-50-01-29s25.tif]

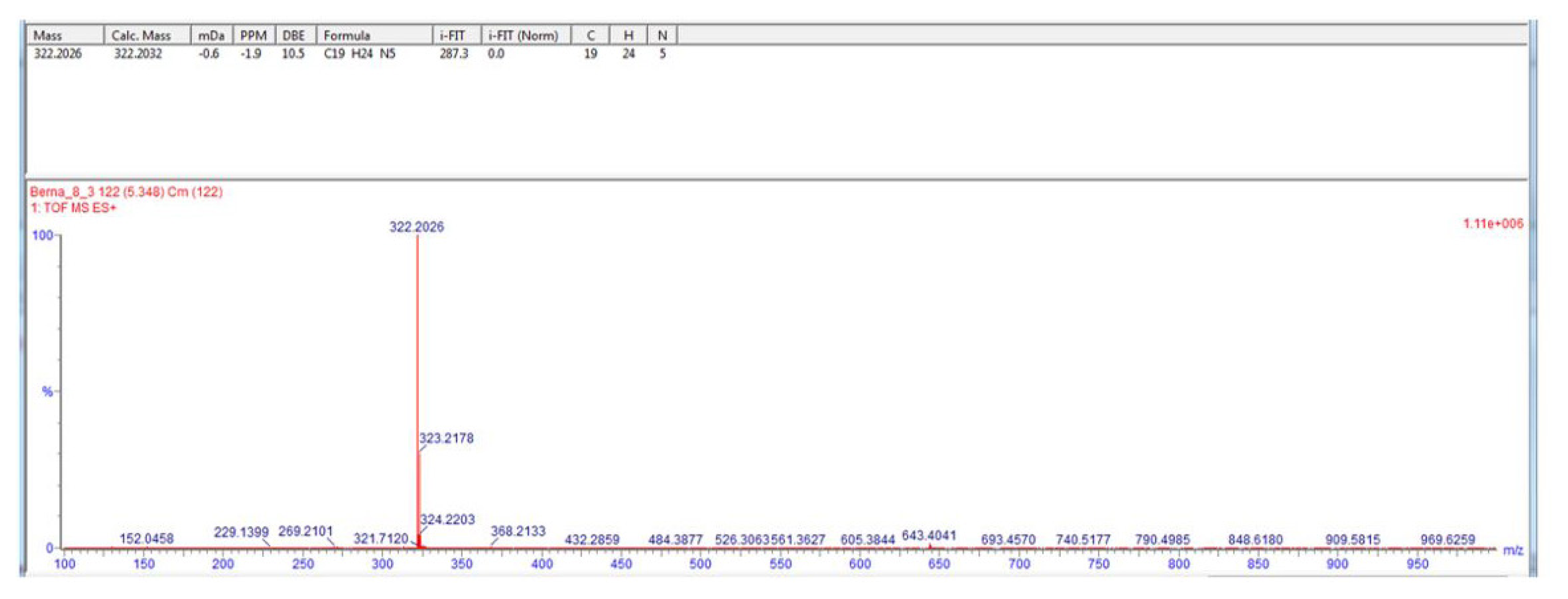

Supplement: Figure S26. — HRMS spectrum of Compound 8. [file tjb-50-01-29s26.tif]

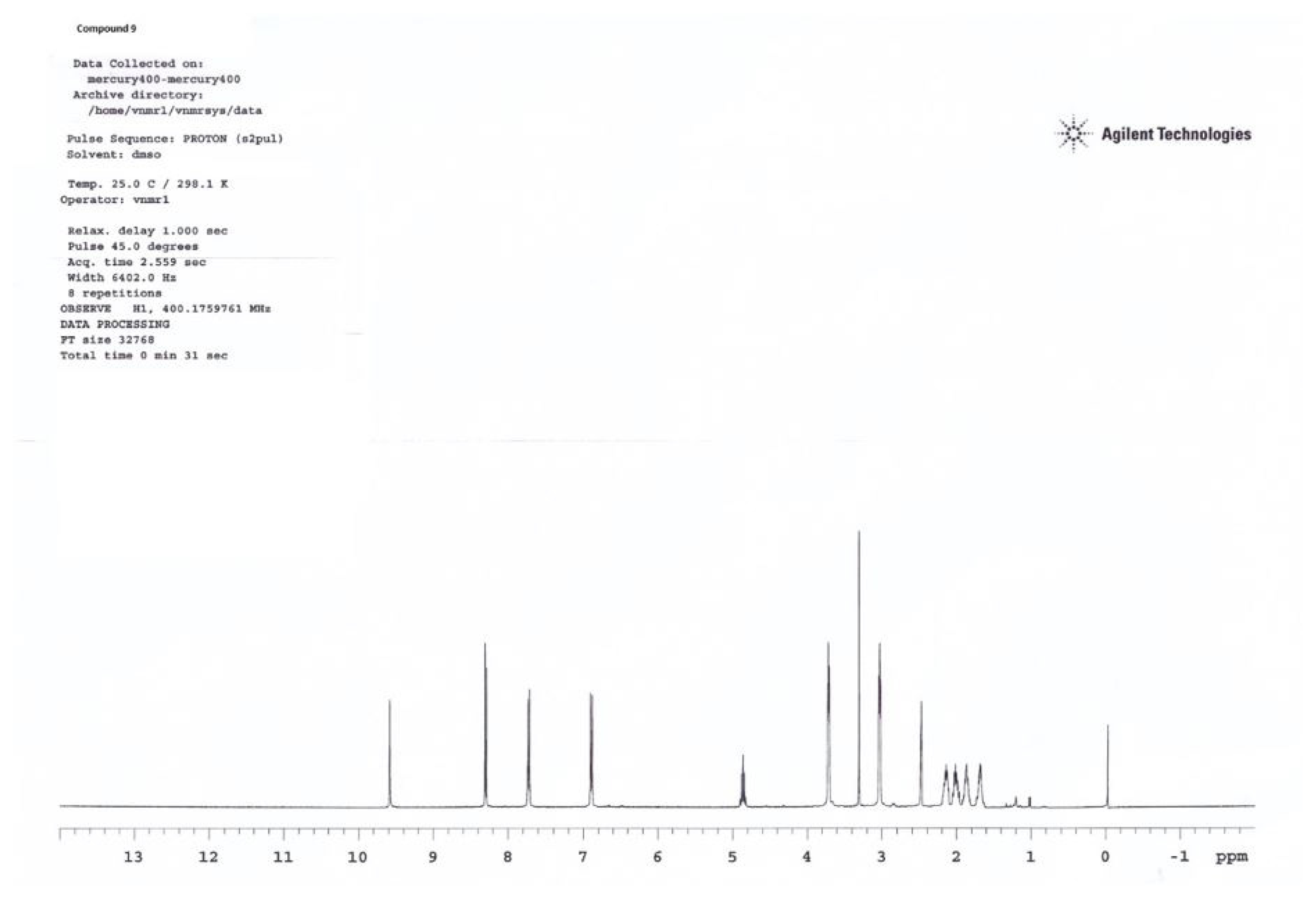

Supplement: Figure S27 — 1H NMR spectrum of Compound 9. [file tjb-50-01-29s27.tif]

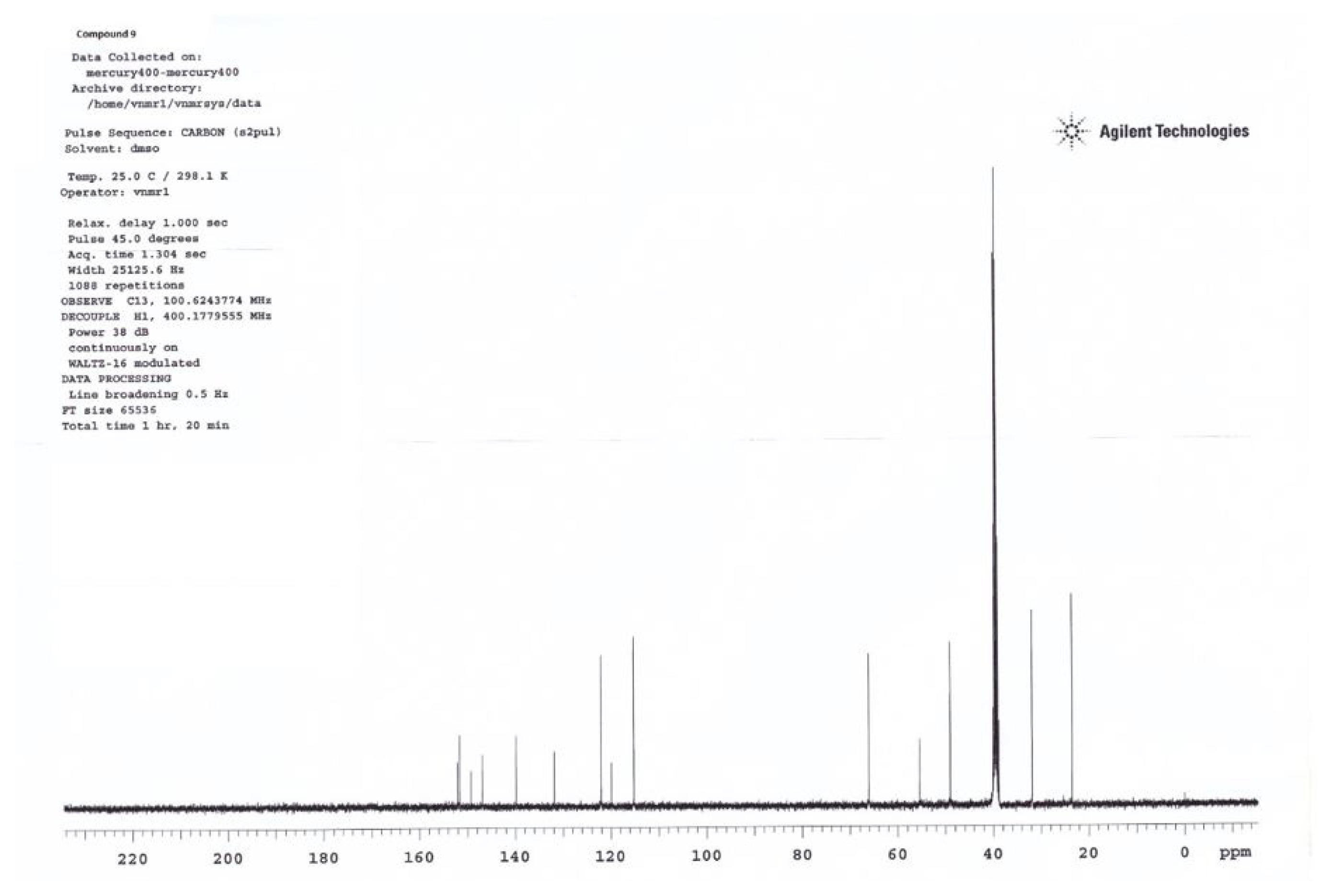

Supplement: Figure S28 — 13C NMR spectrum of Compound 9. [file tjb-50-01-29s28.tif]

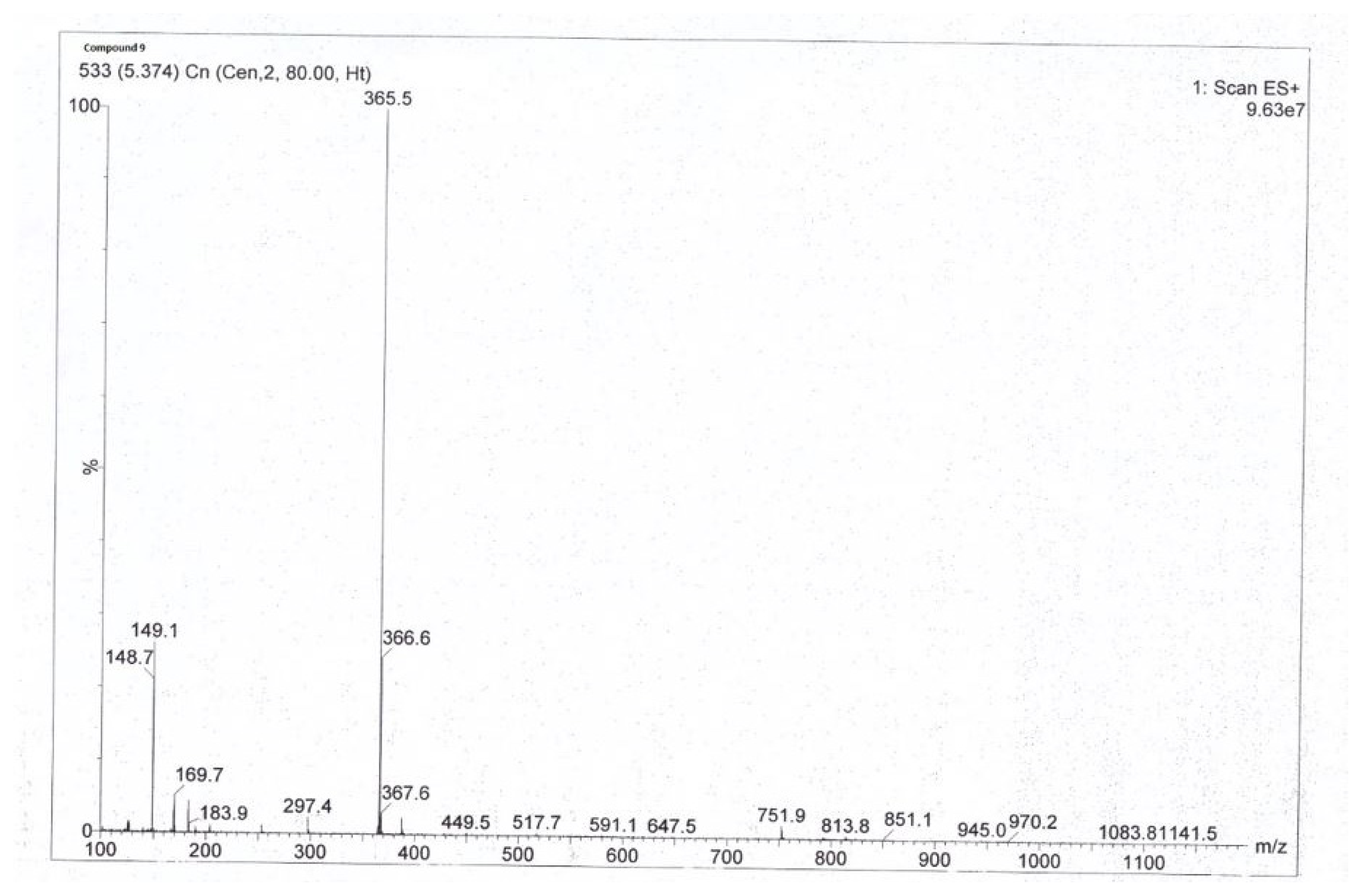

Supplement: Figure S29 — Mass spectrum of Compound 9. [file tjb-50-01-29s29.tif]

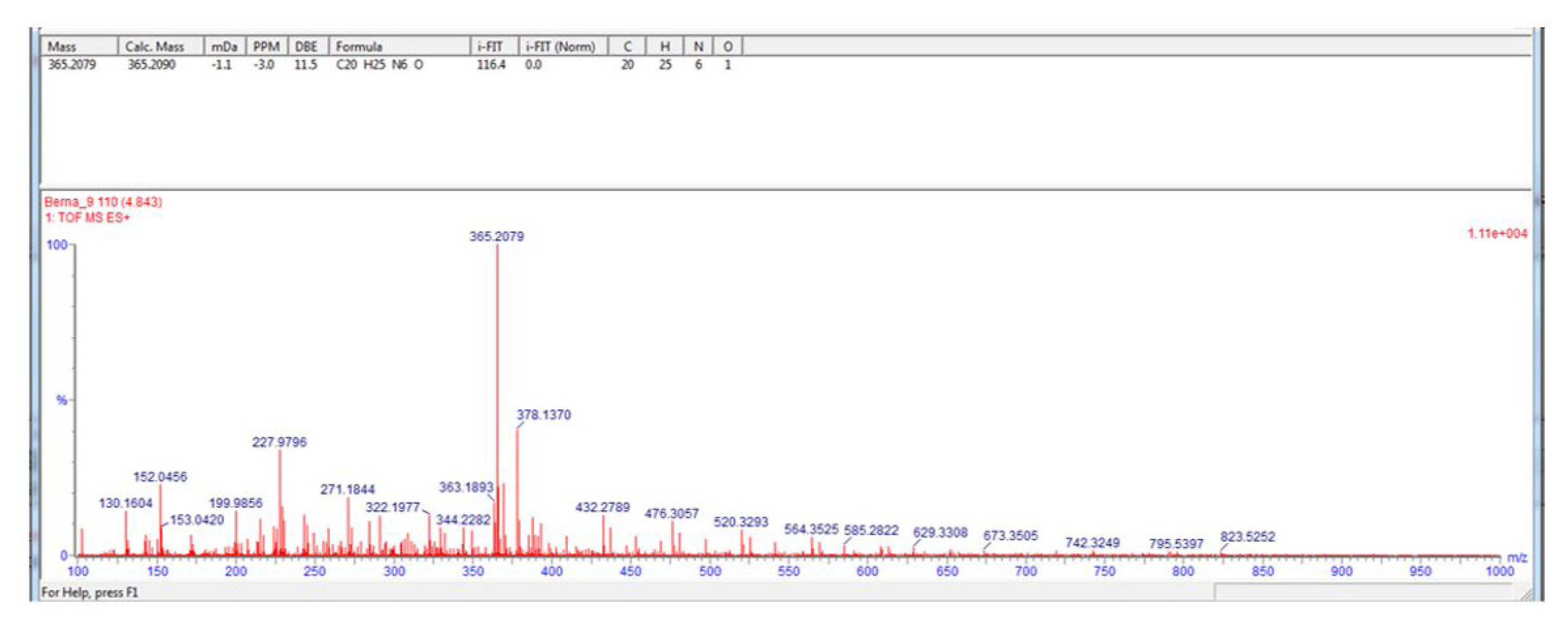

Supplement: Figure S30 — HRMS spectrum of Compound 9. [file tjb-50-01-29s30.tif]
